# Thermographic Phosphors for High Temperature Measurements: Principles, Current State of the Art and Recent Applications

**DOI:** 10.3390/s8095673

**Published:** 2008-09-15

**Authors:** Ashiq Hussain Khalid, Konstantinos Kontis

**Affiliations:** School of Mechanical, Aerospace and Civil Engineering, University of Manchester, England, UK; E-mail: K.Kontis@manchester.ac.uk

**Keywords:** Thermographic, phosphors, temperature, measurement, applications, laser, fluorescence, phosphorescence, luminescence, thermometry

## Abstract

This paper reviews the state of phosphor thermometry, focusing on developments in the past 15 years. The fundamental principles and theory are presented, and the various spectral and temporal modes, including the lifetime decay, rise time and intensity ratio, are discussed. The entire phosphor measurement system, including relative advantages to conventional methods, choice of phosphors, bonding techniques, excitation sources and emission detection, is reviewed. Special attention is given to issues that may arise at high temperatures. A number of recent developments and applications are surveyed, with examples including: measurements in engines, hypersonic wind tunnel experiments, pyrolysis studies and droplet/spray/gas temperature determination. They show the technique is flexible and successful in measuring temperatures where conventional methods may prove to be unsuitable.

## 1. Introduction

This paper aspires to review the current state of temperature measurement using thermographic phosphors including fundamental principles and a survey of recent applications. Many of the techniques utilised in phosphor thermometry are similar in nature to organic pressure/temperature sensitive paints (PSP, TSP) [1]. These have advantages in certain situations, but unfortunately have a modest upper temperature limit, typically no higher than 300 C. Inorganic phosphor materials have much higher temperature tolerances and this review focuses on temperatures beyond the current limit of organic TSPs to around 2,000 K. The review starts with a brief introduction and history of luminescence, which is followed by the generic phosphor thermometry system. Next, the theory and fundamental principles behind phosphor thermometry are described. There are many different ways in which a phosphor can reveal temperature; these different response modes are discussed. A very good review was written a decade ago by Allison and Gillies [2]; thus the present review aims to focus on recent developments in the past 15 years. Later sections review the current state-of-the-art instrumentation/apparatus that are commercially available for a thermographic phosphor system, including detectors and excitation sources. The last section surveys a few applications where thermographic phosphors have been recently used and cited in the literature.

## 2. Historical Background

Luminescence is created from sources apart from heat and is distinct from incandescence and blackbody radiation, or other effects that cause materials to glow at high temperatures. This phenomenon has been observed and reported throughout history. Early Indian and Chinese scriptures dating prior to 1,500 BC refer to light emission from fireflies and glow worms. Aristotle in the fourth century BC observed luminescence from bacteria, fungus and fish and reported the distinction from incandescence: “*some things, though they are not in their nature fire, nor any species of fire, yet seem to produce light*” [3-5]. Nicolas Monardes, in the 16^th^ century, observed blue emissions from a wood extract and great scientists including Robert Boyle and Isaac Newton tried to explain its occurrence; however, it was George Stokes who successfully explained this phenomenon as luminescence in 1852 [5].

Luminescence involves the promotion of electrons to higher energy states with subsequent emissions of light. The 19^th^ century has categorised various types of luminescence, usually dependent on the triggering source of energy. Table 1 illustrates a few examples.

Luminescence induced by light energy is termed photoluminescence and is formally divided into two categories: fluorescence and phosphorescence. Phosphorescence has longer excited state lifetimes than fluorescence; it is usually this that is used for determining temperature in a thermographic phosphor system. Eilhard Wiedemann introduced the term “luminescence” in 1888 to include all light emission including both fluorescence and phosphorescence [6]. The two terms are still open for discussion. Earlier literature refers to phosphorescence for emissions with lifetimes > 10^-3^ s, whereas recent literature suggests lifetimes > 10^-8^ s.

### Phosphors

Phosphors are usually white in appearance and exhibit luminescence when excited. Nowadays they have wide range of applications from CRT tubing, plasma displays. light bulbs and x-ray conversion screens. Alchemists were the first to synthesize luminescent materials, mainly by accident in their attempts to make gold [3, 5]. In 1603, Vincenzo Cascariolo created a material that glowed purple at night having been exposed to sunlight during the day. Later, La Galla in 1612 wrote the first publication on synthetic luminescent material. Another important publication in 1640 termed the word “phosphor” to mean any ‘microcrystalline solid luminescent material’. To distinguish it from the element phosphorous that was later discovered in 1669, long-lived luminescence became known as “phosphorescence” [5]. The synthesised phosphor was probably barium sulphide with a low efficiency. A more stable phosphor was synthesised in 1866 by Theodore Sidot by heating zinc-oxide in a stream of hydrogen sulphide. Soon it was known that these sulphides do not luminance in their pure state, but do when they contain small quantities of activators.

In the 18^th^ and 19^th^ centuries, phosphors were mainly used for detecting invisible particles (UV photons, cathode rays, x-rays and alpha particles) [5]. At this time, with many concurrent advances in other scientific fields such as vacuum science, ceramics, glass working, and electromagnetism, Karl Ferdinand Braun introduced the idea of the cathode ray tube in 1897 and won the Nobel Prize in Physics in 1909 for his contributions [7]. After the introduction of the fluorescent lamp by GEC in 1938, the demand for efficient lighting increased. The need for better CRTs and more efficient lighting accelerated research into properties of phosphors and luminescence.

During the 19^th^ century, Phillip Lenard and co workers synthesised phosphors by firing metallic/rare earth ions impurities (activators) that formed luminescent centres in the host [8]. P.W. Pohl and F. Sietz introduced the configurational coordinate model of luminescence centres, establishing the basis of modern-day luminescence physics [8]. Another figure that helped us understand luminescence is Alexander Jablonski whose work resulted in the Jablonski energy diagram, a tool that can be used to explain the kinetics and spectra of fluorescence, phosphorescence, and delayed fluorescence. Other key figures include Frank Condon and Fonger and Struck, who have helped us to understand certain temporal properties of luminescence.

### Thermographic Properties

Phosphors are thermographic if they exhibit emission-changing characteristics with temperature. The idea of emission analysis for sensing technology is not new. For the case of pressure measurements, the Stern-Volmer relationship between emission intensity and air pressure dates back to as early as 1919. The idea of using phosphors for temperature measurement was first cited in 1937 [9] during the development of the fluorescent lamp where a loss of luminescence was observed with increasing temperature. Although Neubert suggested the idea, it was not until the early 1950's when Urbach and Bradley, cited in Allison and Gillies [2], first utilised phosphors to obtain temperature distributions on a flat wedge. Czysz and Dixson carried out tests producing thermal maps of models in wind tunnels [10, 11]. By applying a phosphor at the tip of an optical fibre, Wickersheim and co-workers investigated many phosphors with a variety of applications and their work led to the commercialisation of fluorescence-based thermometry systems [12]. Grattan and associates also investigated a variety of fibre tip thermometry systems based on a variety of phosphors. Cates [13] and co-workers developed remote measurement systems by adhering a layer of phosphor onto a surface of interest rather than at the end of a fibre [14], offering the system greater flexibility and allowing remote measurements of moving surfaces.

The capture and analysis of fast pulses was mainly nuclear and particle physics territory and required very expensive and sophisticated instrumentation. Over the past two decades, advances in technology, electro-optics and electronics have opened up new techniques and possibilities. Affordable, new detection systems that allow time resolution measurements in the picosecond regime, and newer short pulsed laser systems with increasingly higher pulse energies are being used to devise more elaborate systems that enable greater accuracy and range, opening up newer application areas. Due to the sensitive emission profiles, spatial resolution and high specificity of luminescence, florescence spectroscopy is rapidly becoming an important tool in sensing technology, spanning across many scientific disciplines. It is now very common for biomedical and aerospace applications for detecting oxygen levels, pressure, temperature and even cancer cells.

There are many organisations, joint collaborations and universities that are advancing the field of phosphor thermometry. Examples include Oak Ridge National Laboratory, NASA, Southside Thermal Sciences, Rolls Royce, Pratt and Whitney, University of Lund, University of Manchester, Imperial College and Cranfield University; with key researchers that include: S. Allison, S. Alaruri, B. Noel, M. Cates, G. Gillies, D.L Beshears. A.L Heyes, Seefeldt, J. Feist, T. Bencic and A. Omrane.

## 3. Physical Principles of Luminescence

### 3.1 Basics of Luminescence

This section introduces the fundamental physics of luminescence, later specialising into the luminescence in phosphors. It will attempt to explain various responses that change with temperature, giving phosphors their sensing properties. It will start off with the Jablonski diagram which explains luminescence in general, and later moves on to the configurational coordinate diagram and the charge transfer curve model that helps in the understanding of the sensing properties of thermographic phosphors.

**Figure 1.**
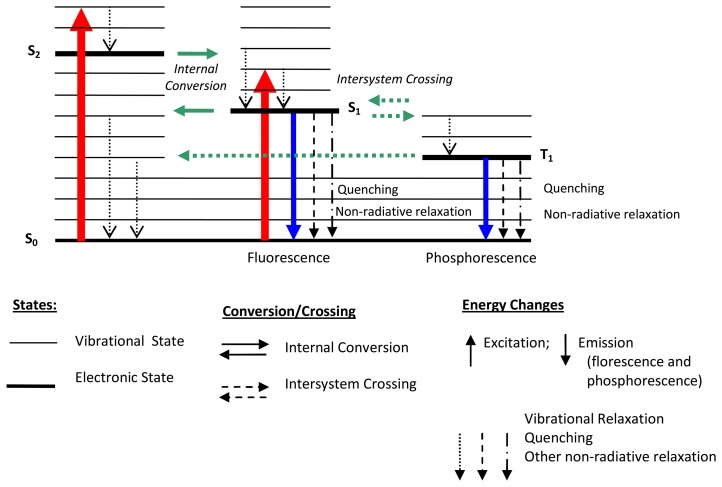
Jablonski energy level diagram showing the luminescence process.

Luminescent processes are governed by a few important events that occur on timescales orders of magnitude apart. In general, excitation causes the energy of luminescent molecules to jump to higher electronic states. However, the configuration does not permanently remain excited. Vibration relaxation, internal conversion, intersystem crossing and emissions soon follow, resulting in the excited state returning back to the ground or an intermediate state. This process can be neatly summarised with a Jablonski energy-level diagram (Figure 1).

For any particular molecule, several electronic states exist. There are a combination of different available orbits (singlet states – S_0_, S_1_, S_2_, ) and spin orientations (triplet/intermediate states – T_1_, T_2_), represented by thick lines, that are further divided into a number of vibrational and rotational energy levels, represented by the thinner lines in Figure 1.

Excitation (e.g. S_0_ to S_1_, S_2_) involves the absorption of sufficient energy to raise a molecule's electrons into electronic states of S_1_ or S_2_. This molecule does not remain excited continually. According to Bell *et al.* [15], the ground state (S_o_) is the only stable state with all other states decaying back to this state. According to the conversation of energy principle, the amount of energy absorbed must be released. This happens via:

- emissions of photons equal to the energy-level difference
- energy transfer via quantised vibrational exchange (phonons) in the material
- other complex energy transfer mechanisms [2].

These energy transfers are further detailed as follows, with typical timescales summarised in Table 2.

#### Vibrational Relaxation

Absorption can cause molecules to be excited into higher vibrational states within an excited electronic state (for example S_1_^level 4^); in this case, the most likely transition will be the relaxation to the lowest vibrational energy level (S_1_^level 0^). This can be seen as vibrations occurring in the crystal lattice, sometimes referred as the emission of phonons in quantum physical terms, so that energy is lost as heat [16].

#### Internal conversion

The lowest vibrational level from a excited state can be converted to the highest vibrational energy state of a lower electronic state (for example S_2_^level 0^ can turn into S_1_^level 5^) This usually occurs when two electronic energy levels are sufficiently close. According to Bell [15], internal conversion results in vibrational relaxation with energy eventually being lost as heat.

#### Fluorescence

This radiative transition from an excited state is accomplished by the emission of a photon. This is generally proceeded from a state of thermal equilibrium to various vibrational levels The emission wavelength, calculated by Planck's equation (dE = hv = hc/λ), is found to be less than the excitation wavelength due to energy level differences, resulting in emissions of longer wavelengths (Stokes shift).

#### Quenching

There are several non-radiative relaxation processes/transitions that compete with radiative processes. One such transition is quenching. This occurs when energy is transferred to another nearby molecule. Oxygen is an effective quencher. The probability of occurrence is dependent on the quenching substance and concentration. By increasing the probability of quenching, the probability of radiative emission (luminescence) will decrease. This principle forms the basis of oxygen and pressure sensitive paints [15].

#### Intersystem crossing

This is a transition from S_1_ to T_1_. Intersystem transitions require changes in electron spin and generally have an extremely low probability of occurrence. According to Turro [17], molecular structure and higher atomic size increases this probability; therefore, molecules containing heavy atoms (e.g. transitional metals) often facilitate intersystem crossing, making these as common as internal conversions. Many efficient phosphors originate from a deliberately added impurity [2]. At this point if the molecule has not returned to its ground state, further possibilities may occur:

- *Phosphorescence* transition to S_0_. This process is orders of magnitude slower than fluorescence. The energy level of T_1_ is lower than that of S_1_ and therefore the emission wavelength of phosphorescence is higher than that of fluorescence.
- Intersystem crossing from T_1_ to S_0_
- Quenching and other non-radiative transitions
- *Delayed Florescence* - This is when there is an intersystem transition back to S_1_. At this point, the entire process of relaxation back to the ground state starts again. If fluorescence occurs after this (from S_1_ to S_0_), this is known as ‘delayed florescence’. This has the spectrum of fluorescence but the time of phosphorescence.

From the description, one may think that every atom has the potential to exhibit luminescence; according to Sant and Merienne [18] practically all existing materials are luminescent. However, luminescent behaviour depends on relative probabilities of alternatives processes by which excited atoms can return to ground state. According to Heyes [16] the persistence of phosphorescence implies that electrons occupy excited energy levels for extended periods. This allows interactions between excited atoms and the surroundings to have an influence on the nature of the emission. Some influences are thermally driven, making them sensitive to temperature.

The Jablonski model is useful for understanding luminescence in general, and is sufficient to explain oxygen quenching behaviour for pressure sensitive paints (PSPs). However, to understand thermographic principles, the chemical nature of the phosphor and the understanding of the configuration coordinate diagram is necessary.

### 3.1 Luminescence in Phosphors

Phosphors can take a number of forms usually consisting of a host material/matrix doped with activator atoms. Many of the materials that fluoresce efficiently are those that originate from a deliberately added impurity [2]. The added activator atoms are usually rare earth (lanthanides) ions or transition metals, seen in Table 3. Other luminescence centres include antinides, heavy metals, electron-hole centres and ZnS-type semiconductors. Thermographic phosphors for high temperature application usually have rare-earth ion centres in ceramic hosts.

Example hosts include:

- Yttrium garnets e.g. Y_3_(Al,Ga)5012 , YAG
- Yttrium oxides e.g. Y_2_O_3_
- Oxysulfides e.g. La_2_O_2_S, Gd_2_O_2_S, Y_2_O_2_S
- Vanadates e.g. VO_3_, VO_4_,V_2_O_7_
- Yttrium/Lutetium phosphates e.g. YPO_4_, LuPO_4_
- Others include: Al_2_O_3_, ZnS:Ag:Cl, LiGdF_4_, BeAl_2_O_4_

The potential number of phosphor combinations is very large.

**Figure 3.**
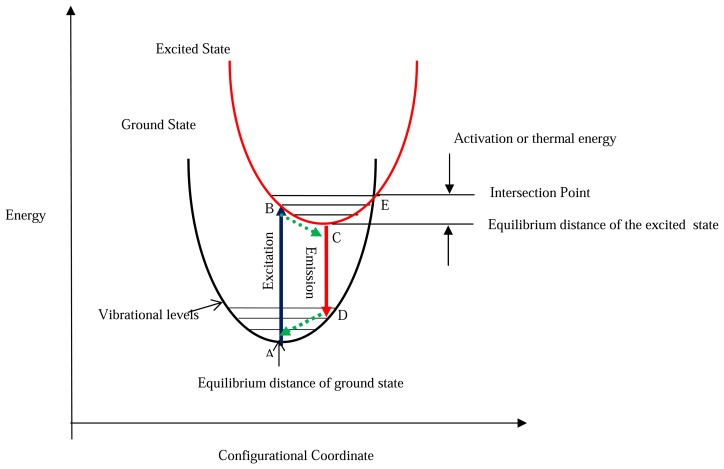
Configuration co-ordinate diagram.

Lanthanide ions, found in the 6^th^ period of the periodic table, are characterised by an incomplete 4*f* shell that is shielded from the effects of the crystal lattice by outer filled shells. Therefore, when a rare earth is mixed into a host lattice in low concentrations it can be treated as a free ion [16]. An example described in Allison and Gillies [2] is that the host material Al_2_O_3_ is transparent and non-fluorescent until Cr^3+^ is added. Luminescent centres are said to be isolated if the dopant concentrations are a few percent [2]. Although this is the case, according to Heyes [16], the host lattice has a profound effect on the thermal response of the phosphor. The influence on the processes of absorption and emission can be explained with the aid of a configurational coordinate diagram (Figure 3). The environment of a luminescent centre is not static and the diagram shows the potential energy curves as a function of a configuration coordinate (deviation from the ion equilibrium distance). Although the model is very simplistic and the shapes of the curves are not parabolic in reality, it shares many features from the Jablonski diagram (Figure 1), and can illustrate several physical phenomena including Stokes Shift. In addition it can also illustrate:

- Absorption and emission band widths
- Understanding of thermal quenching

Like the Jablonski diagram (Figure 1) energy potentials and vibrational energy levels are represented by horizontal lines; similarly, absorption and emission transitions are indicated by vertical lines. After excitation, electrons occupying an upper vibrational level of an excited state (point B) will relax to the ground vibrational level of that state (C) losing energy via the release of phonons [16]. Following radiative emission, the electrons reaching a higher vibrational level of the ground state (D) will further lose energy (phonons) on their return to their ground state equilibrium (A). The difference in excitation and emission energy levels can be seen in the diagram illustrating Stokes Shift.

The Frank-Condon principle states that electronic state transition times are much shorter than vibrational relaxation and therefore assumed to occur in static conditions. Based on this, excitation occurs to vibrationally excited levels of the excited electronic state. According to Royer [19], emissions occur from the lowest vibrational level of the excited state, because relaxation from excited vibrational states is much faster than emission.

According to Heyes [16], at temperatures above 0 K, electrons are distributed over different vibrational levels according to the Boltzmann's law.

(1) $$n_{\text{excited}}={n_{\text{ground}}}^{\left(-\frac{\Delta E}{\text{kT}}\right)}$$

where ‘n’ is the electron population at a given state; ‘E’ is the energy difference between these states; ‘k’ is the Boltzmann constant and ‘T’ is the temperature.

If the temperature is high enough, electrons in the excited state can intersect the ground state curve (point E) allowing vibrational relaxation via phonon release to the ground state without any radiative emission. Ranson [20] describes this as the absorption of thermal energy (phonon) from point C, which excites the electrons to the intersection point E. Since non-radiative processes can now also take place, the observed luminescence intensity from a large quantity of excited ions will diminish, explaining the thermal quenching behaviour that is observed for most thermographic phosphors.

When the temperature is elevated, electrons are spread over a number of vibrational levels in the excited state. Since radiative transitions that can take place between any of the vibrational states in the excited and ground states, a broadening of the of the emission lines is expected [16].

Photo excitation alone can sometimes promote electrons into high vibrational levels at points beyond the intersection point (E) which results in a purely non-radiative emission, with no luminescence being observed. In some cases this may explain why higher energy photons (lower wavelengths) can actually dampen observed luminescence.

An add-on to the configurational coordinate model that explains quenching behaviour in different host materials is proposed by Fonger and Struck [21]. According to these authors, the outer crystal field, which is highly dependent on the chosen host, causes another energy potential (charge transfer state) that can be added on to the existing configuration coordinate diagram (Figure 3). Excited electrons can now return to the ground state via the charge transfer (CT) curve. Suppose an excited electron reaches an excited state of E3; it would normally return to the ground state by radiative emission. However, if the electrons are further excited by elevated temperatures (thermal activation), the electrons can intersect the crossover point of the CT curve, enabling the transfer of electrons to a lower energy level of E2 without any radiative emission. Likewise, electrons in E2 or E1 states can transfer its energy to the ground state in the same way. Different hosts will have the CT curve in slightly different places, thus explaining the different behaviour from various hosts.

## 4. Generic phosphor thermometry system and comparison with other techniques

**Figure 5.**
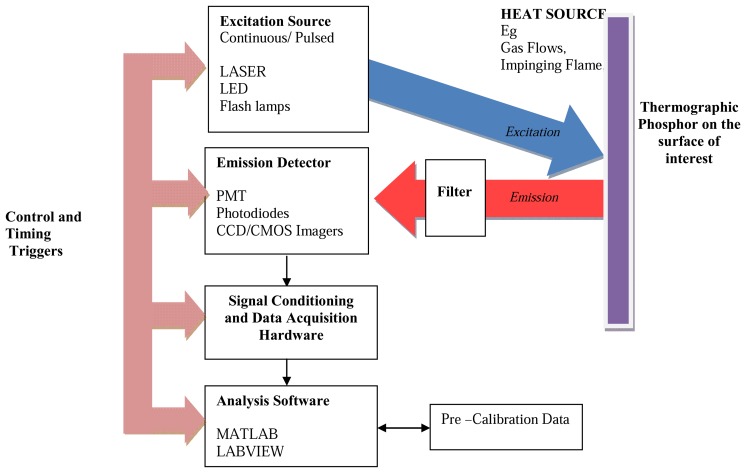
Generic layout for a thermographic phosphor system.

A generic phosphor thermometry system comprises of components illustrated in Figure 5. An excitation source is used to excite the phosphor that is bonded onto the surface of interest. The subsequent emission is passed through an optical filter to separate and filter out unwanted emission wavelengths. The data are stored for later analysis and comparisons with pre-calibrated data to determine temperature. Sometimes, the entire system is controlled by software, such as Labview, that can control the gating time of the detector, the triggering of the excitation source, and sometimes also the heat generating source/phenomena.

The system design in terms of the choice of phosphor, excitation source and detector, will depend on the application and the response mode the user is trying to capture. There are a variety of phosphors each with different responses that can be matched to a variety of different applications. In terms of light sources, intensity methods usually require a continuous beam, and lifetime methods usually require a pulsed source. However, due to increasing blackbody radiation levels at high temperatures, intensity mode researchers are also resorting to pulsed sources since the energy/pulse can be made much higher. For detection, there are a range of choices from point measurement PMTs to CCD imagers.

A comparison of the thermographic phosphor technique to conventional techniques is again dependant on application. There is a mix of characteristics such as accuracy, cost, time, feasibility, durability and intrusiveness, which will ensure some techniques to be more favourable than others. At high temperatures, in excess of 500°C, the environment places severe demands on thermometry apparatus and techniques. Examples of alternative established techniques include the use of thermocouples, RTDs, pyrometry, temperature sensitive paints, liquid crystals and thermal paints. Thermocouples are usually cheap, accurate and easy to install. However, in complex flow conditions, and in rotating environments, such as those experienced in gas turbines, thermocouples can be intrusive, difficult to install with routing of the wires being problematic, and the measurement could lack detail, since it is only provides discrete measurements. In such cases, remote non-contact sensing may be more appropriate. Table 4 highlights some key considerations of alternative technologies in such situation.

Competing non-contact techniques include radiometric infrared thermography and pyrometry. Radiation pyrometry is the current standard for such measurements and offers many advantages over thermocouples including:

- No upper temperature limit since radiation energy increases with temperature
- Fast response and does not have inherent thermal inertia of thermocouples
- Non-intrusive
- Routing problems are reduced
- Immunity to electromagnetic interferences from surrounding environment [23].

Despite these advantages, there still remains sources of error that limit its use. These include: Issues with emittance variation with temperature, reflected radiation and gas stream/flame interference, making them very sensitive to the environment [23]. Phosphor thermometry is largely immune to these errors, allowing it to be used in such environments and other environments where conventional methods prove to be impractical.

Another effective technique used for high temperature measurements, especially in gas turbines, is the use of thermal paints and melts. Thermal paints undergo permanent colour changes as the temperature increases. Thermal melts, containing layers of various metals alloys, can be used to determine temperatures by observing the molten surface. However, this technique requires skill and experience from the operator for accurate measurements. Due to irreversibility, this method can be very expensive, only providing peak temperature information for a single test.

The disadvantage of the phosphor thermometry technique is that it requires the phosphor to be bonded on the surface of interest. The phosphor coating, regardless of thickness, may possess sufficient heat capacity and thermal conductivity to alter its thermal environment, exhibiting a certain level of intrusiveness. This may not be a problem at ambient temperatures, where heat fluxes are low and effects of blackbody and emissivity are negligible. However, at high temperatures, especially in gas turbine environments, it may be necessary to develop a thermal model to determine whether heat transfer will impose a limit to the accuracy of the measurement [2]. Bonding may also be problem if vapour deposition methods are to be utilised, limiting the area that can be coated. Another problem with the thermographic phosphor technique is that there is an upper temperature limit due to increasing blackbody radiation and reducing phosphor signals at higher temperatures. At the moment, the highest temperature recorded is 1,706°C under laboratory conditions [24].

## 5. Different Response Modes

**Figure 6.**
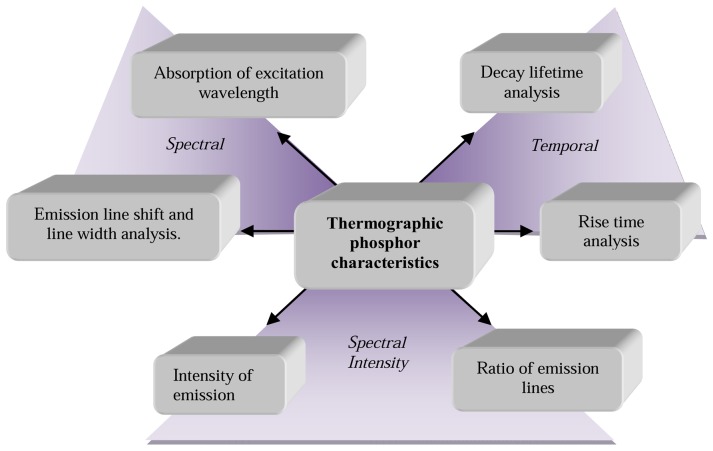
Different response modes for thermographic phosphors.

Temperature can affect the response of a phosphor in several ways. This gives the phosphors their temperature sensing characteristics. This section reviews all known responses that are illustrated in Figure 6.

### 5.1 Intensity Mode

**Figure 7.**
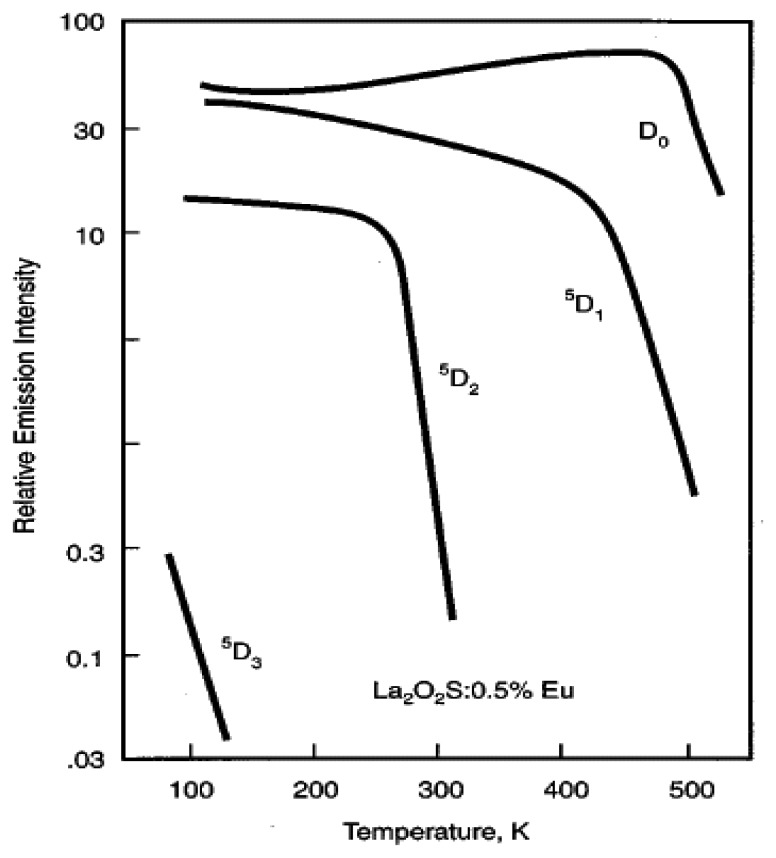
Variation of emission intensity with increasing temperature. Taken from [2].

When a continuous light source is used to excite the phosphor, electron populations are constantly being excited to higher states and returning back to their ground states. An equilibrium level is usually reached, indicated by a steady level of emission intensity. If the temperature is large enough, then probability of deactivation via a non-radiative process is more likely; this will be observed in a reduction in intensity. Various authors have investigated the effects of temperature on intensity for various phosphors and their emissions lines, and this has shown to be true for most cases. Figure 7 shows an example of intensity variations of some emission lines of La_2_O_2_S:Eu phosphor [2]. Most emission lines show a decrease in intensity with temperature. However, there are some emission lines where there is an increase in intensity over a certain temperature range. This may be due to increases in absorption at that wavelength. The will be explained further in section 5.5.

By calibrating the intensity response over a temperature range, temperature measurements can be made. A complete 2D acquisition can be achieved using CCD cameras with each pixel serving as a separate sensor. For a 1MP CCD, 1 million points can be monitored.

A common problem with intensity based techniques is that the observed intensity is also a function of other variables. If they are not taken into account, large errors can remain. Examples of such factors include: non-homogenous illumination, light source instabilities, phosphor coating thickness and densities, distance and detector viewing angle, surface curvature, reflections and shadings. These problems are documented especially in literature relating to pressure sensitive paints. Researchers have attempted to correct for these errors by using by reference imaging and by other mathematical means [25]. However, a better intensity approach that eliminates many of these issues is the intensity ratio approach.

### 5.1 Intensity Ratio

The intensity ratio mode relies on taking a ratio of two emission lines. By doing this a number of errors can be eliminated. In pressure-sensitive-paint (PSP) literature, pressure sensitive paints were added with pressure insensitive reference dyes to make binary paints. The insensitive dye acts as an intensity monitor. Bell *et al.* [15] reports this technique to be the most successful approach for illumination compensation.

**Figure 9.**
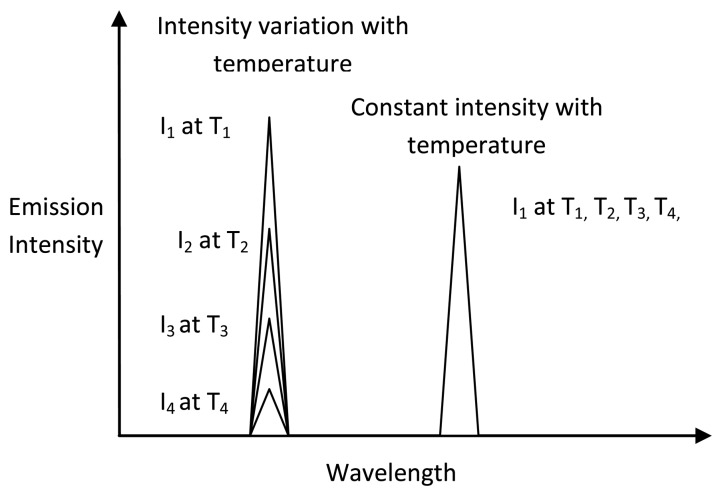
Ideal intensity variations for the intensity ratio response.

The same methodology can be applied to thermographic phosphors. Some phosphors exhibit a multiple emission response with some emission lines being insensitive/less sensitive to temperature. Ideally, the intensity of one of the emission lines should be independent of temperature. Figure 9 shows an ideal intensity variation of the two emission lines with temperature. Phosphors with these characteristics can act as binary paints, and a calibration between the ratio of emissions can be indicative of temperature. It is important that the reference can be excited with the same light and show emissions at different wavelengths so that they can easily be differentiated.

**Figure 10.**
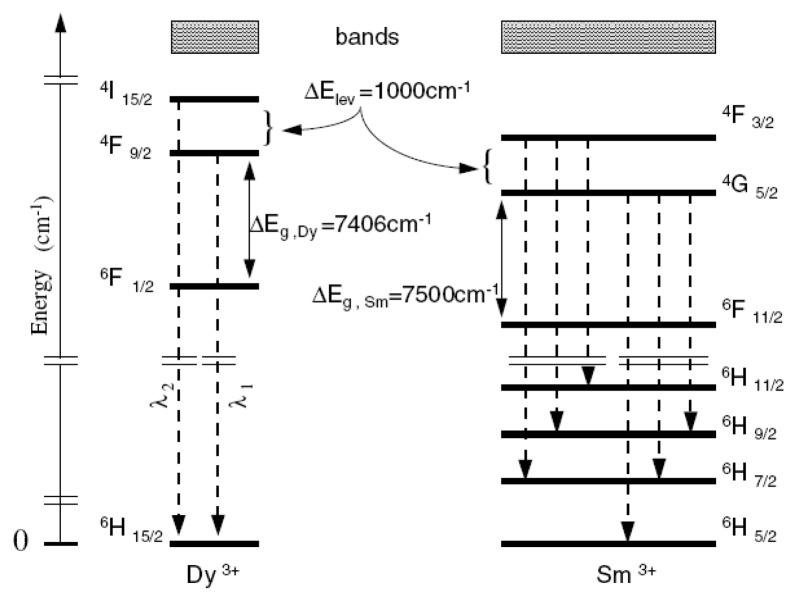
Energy level diagram for free ions of Dy and Sm. Taken from [31] cited in [29].

**Figure 11.**
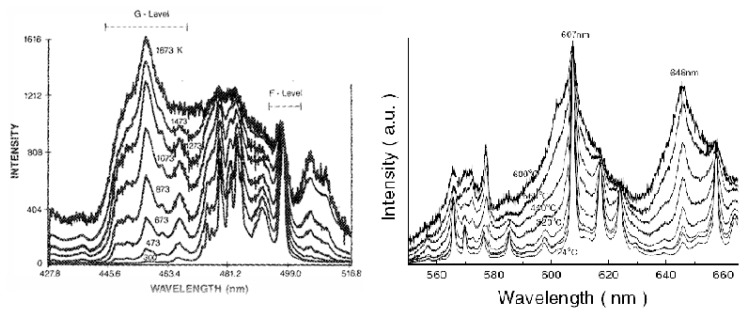
Emission spectra at different temperatures. Left: YAG:Dy [30]; Right: Y_2_0_2_S:Sm [29].

For low temperatures Chyu and Bizzak calibrated a 2D intensity measurement ratio for La_2_O_2_S:Eu to make surface heat transfer measurements for a hot jet impinging on a circular plate [26, 27]. The system reported a range of 292K-333K with an accuracy of 0.5K and repeatability of 0.15K. The cooling effectiveness was also determined from a row of cooling holes [28]. Until recently, dysprosium was the only known rare-earth activator to exhibit an intensity ratio response at high temperatures. Fiest and Heyes [29] showed similar response with samarium-doped phosphors. The main mechanism behind this phenomenon is thermailisation [30]. When two energy levels are closely separated by a difference of approximately 1,000 cm^−1^, the upper level will not fluorescence at low temperatures due to high multi-photon relaxation that quenches the energy. As the temperature increases, the upper level becomes more populated and hence the fluorescence from this level gradually increases. Figure 10 illustrates the similarities between the energy diagram of free Dy and Sm ions. The diagram is only indicative of the physical principles, and in reality there will be host interactions that resulting in variations in the energy levels which could lead to energy splitting, line broadening and shifting [29]. Figure 11 illustrates the emission spectra of YAG:Dy and Y_2_O_2_S:Sm.

For YAG:Dy, the absorbed laser light excites the dysprosium into an excited state which relaxes to the ^4^F_9/2_ level. This level undergoes fast thermal equilibrium and pumps a proportion of its population to the nearby ^4^I_15/2_ level. As the temperature increases, there is a gradual build-up of the population to this level, and hence level of fluorescence. However, above a certain temperature, luminescence slowly begins to decrease due to the charge transfer state (CTS) transitions [32]. The ^4^F_9/2_ level emission (496nm) almost stays constant with increasing temperature, and therefore can be used as an internal reference for calibration for level emission. This allows temperature determination as a relative, rather than a absolute measurement eliminating significant sources of error [30].

These two discrete energy states produce two distinct emission lines. According to Heyes [16], the electron population follows the Boltzmann's relation and is dependant on the temperature and the energy gap. The ratio of the two emission lines can be easily determined by monitoring the increase in fluorescence relative to the lower level.

(2) $$R\left(T,\Delta E\right)=\frac{I_{e}}{I_{g}}=1^{\frac{\Delta E}{\text{kT}}}$$

The intensity ratio technique using thermographic phosphors was first cited in Gross *et al.* [30] using YAG:Dy3 with a reported temperature range of 300K-1,500K and an accuracy of ±9 to ±50K. Kontis *et al.* [32], reported a similar system utilising two gated ICCD cameras. Temperature calibration was made between 295K-1,350K, with a reported accuracy and repeatability of ±2.5K and <0.3%. The system was used for thermal measurements on a ceramic plate exposed to an impinging jet flame [32], and surface heat transfer measurements in a supersonic combustor [33].

Heyes, Feist and Seedfeldt [34] investigated the intensity ratio for dysprosium using YAG and YSZ hosts. Temperature calibration was made between 300-900K, with data repeatability around ±0.6% [34]. The system was used for temperature measurement on ceramic and alloy plates that were heated by flame impingement. YSZ is used for making gas turbine thermal barrier coating; the tests demonstrated the capability of making ‘smart TBCs’ with instrumentation abilities. The same authors have also investigated Y_2_O_2_S:Sm phosphors using the intensity ratio mode between 300-1,100K and showed an uncertainty of ±1%; they also tested the lifetime decay response mode from 900-1,425K and showed an uncertainty of ±1% and 0.1% at higher temperatures [29].

The drawback of intensity ratio response is that two separate detections are required. The conventional way to achieve this is by using two cameras with appropriate optical filters to detected the intensity of the desired wavelength. Another way to achieve this is by using a filter wheel. Table 5 compares these techniques.

More recent approaches include the use of a cube beam splitter to ensure that the images are spatially identical. This approach was used by Kontis [32], with the schematic shown in Figure 10. The total intensity will split two ways and a reduction in the intensity would be expected.

Stereoscopes have also been used. A stereoscope has two apertures which allow two images to be independently filtered using a single camera. It provides similar advantages as the ‘filter wheel’ approach, with the additional advantage of having no moving parts. This approach was adopted by Heyes *et al.* [34] to image the dual emission ratio response of a YAG:Dy and YSZ:Dy. The system was later enhanced to also allow the simultaneous measurement of lifetime decay response. This system allows the cross checking of temperature using the two methods, and also extends the dynamic range of measurement [35]. Similar two mode response systems have been reported by Omrane and Hasegawa [36].

### 5.2 Lifetime Decay Analysis

This method is based on the decay mechanism of phosphor emission. The method is a well established technique for studying emissions of fluorescent molecules, and is used in a number of disciplines. It eliminates many of the issues related with intensity based approaches. The approach is:

- Insensitive to non-uniform excitation
- Insensitive to dye concentrations/surface curvature/paint and thickness
- The approach can be used in high ambient light environments
- The system can take into account photo-degradation [25].

Reponses are usually observed using fast responding detectors, such as PMTs. This method is extremely effective and current detectors can observe decay lifetimes as short as a few hundred picoseconds with single photon counting capability.

Excitation promotes a large number of electrons into an excited state. When excitation is ceased, electrons return to their ground equilibrium level. For simplicity, this is either a radiative or non radiative transition. The rate of the electron population returning to the ground state can be expressed mathematically as:

(3) $$\frac{\text{dN}}{\text{dT}}=\lambda N$$

with the solution yielding to

(4) $$N_{o}e^{-\lambda t}$$

where N(t) is the quantity of electrons at a given time, N_0_ is the initial quantity of excited electrons at t=0, and λ is the decay constant, the rate at which electrons make this transition.

The mean lifetime of which an electron remains in the excited state can be easily calculated.

(5) $$\tau =\frac{1}{\lambda }$$

Since the two transition pathways (radiative and non-radiative) compete and mutually exclusive, the decay constant can be written as the sum of the two possible rates of transitions. The analysis excludes the effects of interaction between activators, impurities in the host that can lead to further processes and change the simple exponential decay signature.

(6) $$\lambda =k_{r}+k_{\text{nr}}$$

The radiative rate (k_r_) is an temperature independent term and can be considered as being a constant, whilst the non-radiative (k_nr_) transition becomes highly temperature dependant after the quenching temperature. For a given temperature, the probability of an single electron taking a transition pathway can be calculated from basic probability theory, resulting in:

(7) $$\begin{array}{ll} \text{Probability of radiative emissions}: & P_{r}=\frac{k_{r}}{k_{r}+k_{\text{nr}}} \end{array}$$

(8) $$\begin{array}{ll} \text{Probability of non-radiative emissions}: & P_{\text{nr}}=\frac{k_{\text{nr}}}{k_{r}+k_{\text{nr}}} \end{array}$$

If the temperature is increased, the decay rate via non-radiative means (k_nr_) also increases. This has the following consequences highlighted in Table 6.

In summary, the probability of radiative transition will decrease whilst the probability of non-radiative transition will increase. By assuming the electron population is proportional to the observed luminescent intensity. The lifetime decay relation can be represented as:

(9) $$I(t)=I_{o}e^{\frac{t}{\tau }}$$

where I_o_ is the initial intensity at time t = 0, and τ is the decay constant.

Figure 13 illustrates typical lifetime characteristics with increasing temperature. The graph shows faster decays with temperature. The relation is only held after the quenching temperature. Researchers have also observed variation in intensity levels with temperature that is not shown in the figure. Figure 14 illustrates the decrease in lifetime decay with temperature for a range of phosphors. It also shows the quenching temperature for some of the phosphors.

Since the lifetime approach is independent of illumination energy, the problems associated with model deformation, movement, shading and uneven light distribution do not exist [25]. In terms of disadvantages, the lifetime method suffers a lack of signal strength [37] as excitation light, in pulsed form, is only available for fractions of the time. To compensate for this, high-powered laser pulses are commonly used. Increasing the pulse strength risks the destruction of the paint. This is true for pressure sensitive paints; however, phosphors have much higher damage tolerances.

The highest temperature recorded using phosphor thermometry was obtained by researchers at ORNL, who successfully calibrated YAG:Dy to 1,705°C using the lifetime decay approach [24].

#### Lifetime Imaging

In the past, the biggest drawback of measuring lifetime decay profiles was due to instrumentation limitations that were only feasible to provide spot measurements. The intensity method, despite its problems, was more attractive as 2D thermal maps could easily obtained using CCD imaging. Previously, distribution maps using the lifetime approaches were built up using point measurements coupled with a XY scanning device. Davies [37] built such as system to determine pressure distributions on a cylinder, and later developed the SUPREMO *(SUrface PREssure Measurement using Optics)* systems for pressure sensitive paints. In recent years there have been many advances in imaging technologies making it practical for temporal responses, such as the lifetime decay response, to be imaged to reveal temperatures of 2D surfaces.

Fluorescence decay lifetime imaging using CCD/CMOS camera has seen much application in the biomedical industry, and was originally developed for oxygen detection in a small area [38]. This system was later modified for wind tunnel experiments [39]. Lifetime imaging using phosphors for thermal measurements has been used intensively in the past few years by a team of researchers at Lund University who claim to be the first to obtain such 2D measurements using this approach [40]. Figure 15 shows an exponential curve fit for a single pixel from a series of images that were carefully triggered at different frames using a high speed camera.

#### Frequency Domain Lifetime Decay

It is possible to determine decay lifetimes in the frequency domain using a specimen excited by a continuous wave. The resulting wave will have a different amplitude and phase due to various time lags of certain luminescent processes. The advantage of this, opposed to a pulsing system, is that luminescent intensity is expected to higher since the phosphor is being illuminated for 50% of the time.

Figure 16 exemplifies the response for different lifetimes, indicating both changes in phase and amplitude. Phase lag is proportional to the lifetime and can be determined; an in-depth analysis can be found in Liu and Sullivan [25]. Burns and Sullivan [41] implemented this technique to map surface pressure measurements. Temperature measurements using phosphors can also be made, and Allison *et al.* [42] reports to have used this technique using blue LEDs.

### 5.3 Risetime Analysis

An investigation by Rhys-Williams and Fuller [43] noted that there are rise times associated with the response of thermographic phosphors. Their research showed that it was dependant on activator concentrations. The phosphor under investigation was Y_2_O_3_:Eu at room temperature. Ranson later analysed risetime characteristics in the late nineties and realised that it could be used for detecting temperature [44].

Ranson *et al.* [45], notes that the crystal structure of Y_2_O_3_:Eu has two sites of symmetry producing energy levels shown in Figure 17. They note the previous work of Heber *et al.* [46] who gives evidence for three potential energy transfers (a,b and c) to level D_0_. The energy transitions of paths ‘a’ and ‘b’ have been observed to be very fast compared to that of ‘c’ [47]. It is this transition that gives this phosphor the rise time characteristics.

The emission of 611 nm (path d) follows the lifetime decay relation shown previously in section 5.2.

(10) $$N(t)=N_{o}e^{-\frac{t}{\tau^{d}}}$$

where N_0_, in this case, is the total number of electrons at D_0._ This is not fixed and depends on the transition paths ‘a’, ‘b’ and ‘c’. The fast transitions ‘a’ and ‘b’ can be modelled as being instantaneous; but the transition of ‘c’ is dependant on the decay of electrons from C_3i_ to D_0_ which decay at

(11) $$N_{c3i}(t)=N_{c}e^{-\frac{t}{\tau d}}$$

Thus, the number of electrons accumulated from path ‘c’ as a function of time is:

(12) $$N_{c}(t)=N_{c}-N_{c}e^{-\frac{t}{\tau^{r}}}=N_{c}\left(1-e^{-\frac{t}{\tau^{r}}}\right)$$

The total number of electrons at D_o_ is then:

(13) $$N_{0}(t)=N_{\text{ab}}+N_{c}\left(1-e^{-\frac{t}{\tau^{r}}}\right)$$

Combining the equations yields the full characterisation of the decay:

(14) $$N(t)=\left[N_{\text{ab}}+N_{c}\left(1-e^{-\frac{t}{\tau^{r}}}\right)\right]e^{-\frac{t}{\tau^{d}}}$$

where τ_d_ = lifetime decay, τ_r_ is the risetime, N_ab_ and N_c_ are the number of electrons by transitions a, b and c, respectively.

The investigations were carried out were carried out using Y_2_O_3_:Eu phosphor with approximately 3% Eu concentration. Previous investigations by Rhys-Williams and Fuller [43] noted that rise times ranged from 60 μs at 5% mole concentration to 320 μs at 0.27% mole concentration. Recent work by Allison *et al.* [48] underwent investigations at 0.5% Eu. The results shown in Figure 18 clearly demonstrate the effects of temperature on risetime, showing a noticeable clear decrease in risetime due to increasing temperatures. Another temperature related response, that is further discussed section 5.5, is also shown; there is an increase in luminescence strength due to increasing temperatures. According to Allison *et al.* [48], this is due to increased phosphor absorption at the excitation wavelength (337 nm nitrogen laser).

### 5.4 Line Shift/Width Method

According to Gross *et al.* [30] temperature can cause the crystal lattice containing the rare-earth to vibrate creating a changing crystal field that produces a broadening of emission linewidths. Frequency shift of the spectral lines can also occur due to thermal expansion of the crystal lattice [30]. Both lines shift variation and broadening can be calibrated to reveal temperature. However, these effects are usually small. The variation in the line shift at 1,000K is only 3 nm, making the sensitivity very small and difficult to detect [16]. Kusama *et al.* [49] utilised this approach for measuring temperature using Y_2_O_2_S:Eu phosphor and the following graph shows variations at ^-^15ºC and 72ºC. Kusama *et al.* [49] suggested quadratic shift according to the equation:

(15) $$\text{E}=\text{A}+{\text{BT}}^{2}$$

where ‘E’ is the expected energy, ‘A’ is the ground energy at 0K, ‘B’ is a constant, and ‘T’ is the temperature in Kelvin.

### 5.5 Absorption /Excitation Bands

Various studies have shown a variation in the excitation and absorption band of some phosphors due to changes in temperature. When a nitrogen laser (337nm) or third harmonic Nd:YAG laser (355 nm) is used to excite a Y_2_O_3_:Eu phosphor, there is a gradual increase in the emission intensity with increasing temperature. According to Allison and Gillies [2], this is because the absorption, at these wavelengths, is weak at room temperatures, and slowly increases with temperature. The absorption spectra for Y_2_O_3_:Eu at room temperature is shown in Figure 20, illustrating the weak absorption lines at 337 and 355 nm. Figure 21 illustrates the shift in absorption band due to increases in temperature. If a linear trend is assumed, it seems reasonable to use these wavelengths for higher temperature detection.

## 6. Other factors

This section reviews other factors that can influence emissions from a phosphor.

### 6.1 Activator concentrations

It has been shown that the activator concentration affects the temporal decay profile and the intensity of the emission. Y_2_O_3_:Eu concentrations less than 5% leads to strongest lines of shortest wavelength [16]. Greater concentrations lead to dispersion with no sharp lines being observable. With increasing concentrations, the energy gap between lines is reduced so electrons reach lower levels from neighbouring ions by non-radiative means. Allison and Gillies [2] notes that higher activator concentrations may alter the fluorescent decay so that it follows a multi-exponential rather than a simple exponential profile, making measurements more difficult to characterise and prone to errors.

As previously discussed, the risetime of the phosphor's response is also affected by the activator concentration. Reducing the dopant concentration increases the rise time for Y_2_O_3_:Eu phosphor [43]. Not much information is available to see whether this is universally true for other phosphors.

It most applications, it can be assumed that thin coatings of the phosphor exhibit the same temperature as the surface of interest. However, in some applications, where temperatures are changing at fast rates, knowledge of the phosphors thermal response is required to properly unfold the temperature [24]. In YAG phosphors, increasing the dopant concentration reduces the thermal conductivity. Kontis [32] notes that most 1% dopant YAG phosphors has a thermal conductivity of 4 Wm^-1^K^-1^, which is reduced to 2 Wm^-1^K^-1^ when the concentration is increased to 3%.

### 6.2 Saturation Effects

High excitation energies can lead to luminescence saturation. This is where the luminescent intensity does not change with increasing energy from the source. In fact, above a threshold, there have been reported cases where luminescent intensity actually decreases with faster decay profiles. There are a number of explanations for this. The laser beam can induce an increase in temperature [50]; in this case thermodynamic consideration must be given for these beam related effects. According to Allison and Gillies [2], this is probably due to the increased probability of two ions being excited in close proximities. This increases the chances of the energy being transferred from one ion to the other, with only one photon being emitted instead of two.

### 6.3 Oxygen quenching / Pressure

Pressure sensitive paints respond to both thermal changes and changes in the level of oxygen. Thermographic phosphors were originally thought to be independent of oxygen changes. Recent investigations are challenging this assumption. These investigations are important if phosphors are to be utilised in areas where the partial oxygen level is likely to change e.g. consumption of oxygen in combustion chambers.

Feist *et al.* [51] investigated the oxygen quenching of Y_2_O_3_:Eu and YAG:Dy. Volumetric percentage of oxygen was changed from 21% to 5% by flooding the furnace with nitrogen. No absolute changes were noted, but the readings resulted in increased uncertainties in temperature measurement. For Y_2_O_3_:Eu, the uncertainties, due to changes in oxygen, were an order of magnitude greater than uncertainties at fixed concentrations, providing a convincing case for oxygen quenching. However, for YAG:Dy, the uncertainty was the same order of magnitude, and so the results for this case may be considered as being inconclusive.

A more recent investigation by Brubach *et al.* [52] showed the effects of various gas compositions on three different phosphors. The results show that variations in oxygen, nitrogen, helium, carbon dioxide, water vapour and methane concentrations do not influence the decay time of Mg_4_FGeO_6_:Mn and La_2_O_2_S:Eu phosphors. (Figure 22 a,b). These phosphors are only influenced by thermal quenching and are suitable for environments where changing gas environments are expected. Y_2_O_3_:Eu (Figure 22c) however showed high sensitivity to oxygen.

Apart from pressure causing an increase in partial oxygen levels, there is also evidence that application of pressure/strain can affect luminescent properties of thermographic phosphors. This phenomenon is not very well understood but becomes very relevant when extreme pressures are concerned. The application of pressure can be viewed as the imposition of compressive strain that can result in changes in both chemical bonds and atomic level orbital configurations. The decay time of Gd_2_O_2_S:Tb decreased by an order of magnitude with application of 2 GPa, while the decay time of La_2_O_2_S:Eu increased by an order of magnitude with application of 3.5 GPa [2].

Although some phosphors may not exhibit oxygen sensitivity, for example La_2_O_2_S:Eu [52], they may possess pressure sensitivity; it is important that both parameters are treated independently. However, in most flow conditions, it is unlikely that these sorts of pressures will be reached (1GPa = 10,000 Bar). Although, Brubach *et al.* [52] investigations showed no change in lifetime for La_2_O_2_S:Eu up to a pressure of 10 Bar (1 MPa), the results presented in

Figure 23, illustrates the decrease in decay lifetime at higher pressures (0 – 50 MPa). In very harsh flows, such as those experienced in gas turbine engines, the maximum pressure is around 50 bar (5 MPa), and the effects of this phenomena may become relevant.

Y_2_O_3_:Eu phosphor showed sensitivity to oxygen quenching and showed irreversible changes after the absolute pressure was increased to 6 Bar [52]. According to these findings, Y_2_O_3_:Eu, which has been a very popular choice of phosphor for turbine engine thermometry, is unsuitable for environments where the pressure and oxygen level is expected to change.

### 6.4 Impurities and Sensitizers

Impurities in the phosphor can affect luminescence. In a simple case, excitation energy acts directly on the activator, as shown in Figure 24, which consequently produces radiative emissions with some energy being lost by other non-radiative means. Impurities in the host material can change atomic electronic environment experienced by the activators. Transition metal impurities, even at low concentrations (1 ppm), can decrease luminescence due to them extracting energy that would otherwise be used to produce radiative emissions. A representation is shown in Figure 25. Since there is a change in the probability of non-radiative and radiative energy transfers, the decay rate of luminescence is also expected to be altered.

It is possible for the energy transfer to act in the other way. UV radiation on impurities can further excite the activator by energy transfer. These added impurities are termed sensitizers if their presence increases luminescence. In some cases, the activator only produces radiative emissions with a sensitizer is present. (Figure 26– case A). The host lattice can itself act as a sensitizer, for example YVO_4_:Eu^3+^. In other cases, both the activator and the sensitiser can be directly excited, (case B), and the sensitizer can also be luminescent (case C). The sensitisers could be additional activators, which further complicates the analysis. Some experiments have revealed that the addition of small amounts of other activators, such as Dy and Tb or Pr, to Y_2_O_3_:Eu decreased the lifetime decay by a factor of 3, with little change in the quantum efficiency [2].

The energy transfer from the sensitizer to the activator is termed Resonance Energy Transfer (RET). RET is also possible when the emission spectra of the sensitizer (donor) overlaps the absorption spectra of the activator (acceptor). The transfer is manifested by the quenching of the donor and the increased absorption from the activator that consequently results in increased emissions. These complex mechanisms can be used to explain risetimes and complex multi-exponential decay profiles.

### 6.5 Particle size

There have been a number of studies to suggest that lifetime decay and intensity changes with phosphors particle size. Investigations into nano-crystalline and coarse grain particles of Y_2_O_3_:Eu phosphors reveal that the excited state parabola on the configuration coordinate diagram may be affected. Konrad *et al.* [54], explains there is an increasing slope of the excited parabola with reducing particle size, shown in Figure 27.

This results in the intersection point between the excited and ground state being increased. Consequently, the quenching temperature is expected to be higher and the lifetime decays are expected to last longer. Work by Christensen *et al.* [55], has shown an increase in lifetime ranging between 436-598 µs, due to reductions in particle size from 0.42 to 0.11 µm. As different preparation and surface bonding techniques produce particle sizes, it seem reasonable to assume that the decay lifetime is not absolute, and therefore it is important that calibration is deployed for that type of preparation or bonding technique.

## 7. Bonding Techniques

Adhering the phosphor to the surface of interest is vital for the successful application of phosphor thermometry. The method should be durable and capable of surviving the exposed environmental conditions, including the maximum operating the temperature. The method should be inert and should not change the spectral and thermographic properties of the phosphor. The phosphor coating should ideally be non-intrusive to the temperature measurement, and therefore provide good thermal contact, which becomes very important, especially when thermal transients need to be measured. This section reviews various bonding techniques that have been used at high temperatures.

### 7.1 Chemical Bonding

This process involves mixing powdered phosphors with chemical bonding agents to create a paint that can be either brushed or air-sprayed on to a surface. The nature of the binder will depend on the surface and the operating temperature range. Epoxy binders have a temperature limit that is reached at a few hundred degrees. Apart from survivability at higher temperatures, chemical binders must allow transmission characteristics that enable the phosphor to be excited and emissions to be detected. Chemically bonded phosphors usually require curing by raising the temperature to 700°C and slowly bringing it back down to room temperature. In the past few years, a variety of commercially available binders have been investigated [56-58]. Propriety binders manufactured by thermal paints experts at Rolls-Royce Plc have been successfully tested up to 1,100°C [16]. Some of the higher surviving commercially available binders include ZYP-ZAP and Coltronics-Resbond, which have shown survivability and fluorescence detection up to 1,600°C [59]. Table 7 compares some of these binders. Goedeke *et al.* [59] notes that although ZYP-ZAP has stronger survivability, the observed fluorescence is higher in Resbond at 1,500°C.

Problems with chemical binders include the possibility of changing the phosphor's atomic configuration, and hence luminescence and thermographic properties. Ideally, chemical binders should suspend the phosphor without changing atomic properties.

Problems associated at high temperatures include differences in thermal expansion that causes the paint and substrate to expand at different rates. According to Allison *et al.* [56], one of the most challenging surface for bonding is high strength nickel alloy due to high differences in the thermal expansion coefficients. At high temperatures, this causes the paint to flake off. To increase the thermal conductivity of the paint, to reduce thermal shock and increase survivability of the paint, tests were conducted with the addition of MgO_2_ in the binder.

Another problem associated with chemical binders is the effects due to thermal exposure. Figure 28 demonstrates the reduction in emission intensity of Y_2_O_3_:Eu phosphor with Resbond 793 binder at 1,400°C after 4 hours of thermal exposure [59]. Similar results were reported by Ranson *et al.* [60] using a different chemical binder. The results showed a reduction in intensity to approximately 10% of their initial value following thermal exposure at 1,200°C for two hours. The reason for this could be simply due to the paint layer flaking off. Other cases report the paint transitioning to a yellow/brown colour, and is still unclear whether it is problems with the optical transmission of UV, or the optical passing of emissions, or a combination of the two that is responsible for the reduction in intensity. Thermal exposure for long periods may force chemical reactions within the phosphor changing its characteristics.

Chemical binders allow the use of spray painting. The advantage of this is that large areas of various sizes and shapes can easily be covered. However, maintaining a uniform surface and controlling the thickness and roughness can be difficult. Tests indicate a variation in intensity across different test pieces [61].

The disadvantage of having binder paints is that the minimum coating size that can be produced is around 10 µm (typical is around 30-60 µm). This is relatively large compared to the vapour deposition and plasma spraying techniques. Greater thicknesses provide greater thermal gradients between the phosphor coating and substrate, constituting to a greater error in measurement.

### 7.1 Vapour Deposition

In this process, a coating is applied by vaporising the phosphor and allowing it to condense on the surface of interest. There are a variety of ways this can be achieved including electron beam (EB-PVD), pulsed laser disposition (PLD), chemical vapour deposition (CVD) and RF-frequency sputtering. No chemical binders are required and therefore there is no interference or problems concerning optical transmittability of UV and emission wavelengths. The resulting coatings are very robust and long-lived with fluorescent intensity being constant throughout its life. They can be made very thin compared to chemical binder paints, and can be finely controlled to have a uniform surface finish. However, the equipment required to produce these coatings can be very expensive and the coatings areas are usually very limited.

During vapour deposition, dopant atoms can be situated in a variety of positions and rotations within the hosts crystal structure and therefore experience a variety of crystal field effects, leading to weaker and wider spectral emissions. Post annealing is required to realign the ions to restore crystalline quality and increase luminescent intensity. Allison *et al.* [2] notes that the high temperatures generated during vapour deposition can irreversibly break down some phosphors, such as oxy-sulphides, and post annealing will offer no benefit.

Ranson *et al.* [60] investigated thin coatings (0.1 µm – 3 µm) produced by RF sputtering and thick coatings produced by binder paints. They found that emission intensity was related to coating thickness, up to a certain level. It was shown that 0.7 µm post annealed coatings provided the same level of intensity as thick coatings (approx 10 µm) produced by chemical binders. These tests were undertaken for Y_2_O_3_:Eu phosphors, and whether this is true for other phosphors is yet to be investigated.

While thick coating produced by binder paints show declining intensity with thermal exposure, Ranson *et al.* [60] showed that thin coatings produced by vapour deposition show no decline in intensity level. Figure 30 compares shows the variation in luminescent intensity as a function of time hours of constant thermal exposure at 1,200ºC. The results show that the binder paints showed a very quick decline in intensity, whilst thin coatings produced by vapour deposition showed no change in intensity.

### 7.2 Flame /Plasma Spray

Flame spraying is portable and applicable to objects of diverse geometries, but has a lower impact velocity than plasma spray [1]. Plasma spraying involves injecting powdered phosphors into an electrical discharge to liquefy them. The technique allows robust, well-adhered coatings made up from a multiplicity of solidified droplets [16]. It is usually used to produce thicker films than vapour deposition methods and can produce coatings exceeding a millimetre. Like vapour deposition, annealing is also necessary to restore crystalline quality and improve luminescent efficiency.

## 8. Consideration factors for high temperature measurements

If a system is used at high temperatures, there are certain factors that will make detection difficult. Many phosphors have reducing intensity whilst exhibiting faster decays. An upper temperature measurement capability will be reached when the phosphor signals eventually becomes too weak relative to the noise inherent in the detection system. There is also increasing blackbody radiation that will eventually become too large making it difficult to separate from the phosphor signal. This radiation can be predicted using Planck's radiation law, and Figure 31 illustrates the increasing amount of radiation as function of wavelength at various temperatures.

There are a number of approaches that can be adopted to reduce these effects and maximise the performance of the measurement system. The use of interference filters at the peak emission wavelengths to filter out the blackbody radiation. The amount of detected radiation is an integration of both the blackbody radiation and phosphor emission. If a large band filter is used, this radiation can still be large in comparison to the spiky emission from the phosphor. Figure 32 demonstrates the amount of blackbody radiation that will be detected if wide and narrow band filters are used. As shown, the use narrow band filters at the precise peak of phosphor emission can reduce the proportion of blackbody radiation passing through the filter, yielding better phosphor signal to blackbody radiation ratios, and hence overall SNR. The downside to this is that there is usually a reduction in the amount of light passing through to the detector, and therefore a compromise will have be made between the choice of filter and the system's ability to detect lowlight level changes. Another complication is that the phosphor's peak emission wavelength may vary with temperature, and a very narrowband filter may not detect the emission at all temperatures. Emission spectra at various temperatures will help decide the best choice of filter.

For any given temperature, the background radiation in is higher at longer wavelengths as seen in Figure 31. Therefore, it is more effective to use phosphors whose emission wavelength is as low as possible. Ranson [20] notes that the level of blackbody radiation at 544 nm, the peak emission of YAG:Tb, is a factor of approximately 5x less than 611 nm, the peak emission for Y_2_O_3_:Eu; Allison and Gillies [2] notes that at 488 nm, one of the peak emissions for YAG:Dy, has an order of magnitude less blackbody radiation than the peak for Y_2_O_3_:Eu. Figure 33 illustrates the intensity that would be required to maintain the same level of signal to blackbody ratio for each phosphor. The quantum efficiency of the phosphor at given temperatures must also be considered. Ranson [20] notes that the strong intensity exhibited by Y_2_O_3_:Eu outweighs the advantage of YAG:Tb in terms of blackbody radiation.

Phosphor emissions can be increased if more energy is put into the system, by increasing the excitation energy. At some point, it is expected that luminescence may saturate, and show no change in luminescence intensity with increasing energy. Further increases may actually reduce the luminescent intensity (Section 6.2). In either case, it seems reasonable to find the peak excitation energy that maximises intensity. In many optical laser systems, this peak is usually not met, and for this reason, a relatively high energy pulsed light source (usually laser) is more suited.

Apart from the increasing blackbody radiation at higher temperatures, there are other limitations in temporal approaches, such as the lifetime decay approach, that will cause an upper temperature limit due to the systems incapability to measure fast decays. The key contributors to this include:

- The limits of data acquisition sampling resolution is reached
- The detectors response time exceed
- Excitation pulse fall times can interfering with the decay time of the phosphor. The energy from a laser is relatively large and even through high optical density narrow band filters are used to block any reflected laser light, some light usually leaks through. If the luminescent decay lifetime is on the same order of magnitude as the fall curve of the laser pulse, then it may be difficult to discriminate between the two. The ideal pulsed light source should have very fast fall times.

## 9. Emissions Detection

This section reviews various different detectors that are commercially available that can be used for luminescence detection. It is split into two sections; the first section looks at point detection, and is followed by imaging.

### 9.1 Point Detection

For point measurements, there are a number of detectors that can be used, with the main ones being PMTs, silicon and avalanche photodiodes, and newly developed Si-Photomultipliers. This section compares these, and the main findings and typical characteristics are highlighted in Table 8.

#### Photomultiplier Tube (PMT)

The PMT has been the most widely used instrument for phosphor emission measurements. They are very sensitive and responsive, with typical rise and fall times in the 1ns regime. The principle of operation is demonstrated in Figure 34. Photons strike a photo emissive cathode which emits electrons that are collected at the anode. These electrons are then accelerated towards a series of additional electrodes (dynodes) that are maintained at a higher potential, generating additional electrons. This cascading effect creates 10^5^ to 10^7^ electrons for each photon hitting the first cathode. This amplified signal is finally collected at the anode where it can be measured. PMTs have large detection areas and can offer a high gain and superior SNR compared to its competitors.

#### Micro channel plate PMT

A MCP-PMT contains an electron multiplier consisting of an array of millions of glass capillaries fused into a thin disk less than 1mm thick. MCP-PMTs are very fast. The time between the generation of the primary emissive electron at the cathode and the arrival of the corresponding bunch of electrons at the anode is very small, with response times in the region of 100 picoseconds, making them around 10X faster than conventional PMTs. In the past MCPs were only available for the detection of VUV, soft X-ray photons and neutrons. They have now been engineered for visible light detection [62].

#### Photodiodes

These are semiconductor light sensors. They feature excellent linearity with respect to incident light, have wide spectral response, are compact, mechanically rugged and have a long life. Response times typically vary from hundred nanoseconds to a few microseconds, making them slower than PMTs. However, recent developments enable them to operate at similar bandwidths.

The signal generated by photodiodes is very small relative to noise inherent in the system, resulting in poor SNR, especially when they are operated at high bandwidths or low light levels. In order to detect lower light levels, it is usual to increase the gain by increasing the feedback circuit resistor value. This has unwanted consequences lowering response speed and increasing thermal noise [63]. A high-speed preamplifier can ensure a wide response speed and lower noise. Avalanche photodiodes have some intrinsic gain and offer lower noise characteristics than standard electronic amplification, making them more suited to lower light conditions [63]. The shot noise is often higher; therefore SNR is not usually improved. However, for low-level detection, gain can be increased to improve SNR, whilst maintaining response speed, until shot noise reaches thermal noise level [63].

The quantum efficiency of these devices is much higher than that of PMT. However, these detectors have much smaller detection areas, and it is likely that more light will be lost in collection optics than gained by quantum efficiency.

#### Si Photomultipliers (SPM)

These are relatively new solid-state devices and have had considerable amount of research over the past decade. Their performance is superior to that of standard and avalanche photodiodes in terms of sensitivity, and is approaching that of PMT detectors [64-66]. In many applications, their performance surpasses that of PMTs. SPM detectors have a number of advantages over PMTs including: small size, low bias voltage operation, magnetic field insensitivity, a higher degree of robustness and immunity to damage from high light condition overexposure. The core of these devices are arrays of APDs operated in Geiger Mode. This technology is thought to eventually replace conventional PMTs. Further details can be found in the references.

### 9.2 Imaging

#### CCD-Charge-Coupled Device

CCDs contain photosensitive elements called pixels that converts photons into charge. The quantum efficiency for these devices can be as large as 90% for back illuminated devices. Conversely, the full-well capacity indicates the upper limit can be detected before electrons spill into neighbouring pixels, smearing the image. In phosphor thermometry, this will be an important factor, since the level of blackbody radiation becomes increasingly intense at higher temperatures.

Phosphor thermometry using intensity-based methods can be relatively straight forward, where fast transfers are not required. Fast transfers will be essential for unsteady cases, or when using temporal approaches. CCDs contain vertical and horizontal registers and an output section. It takes time to read the charge off the CCD and can be approximated by clocking speeds (10-50 million pixels per second). Noise is proportional to clocking speeds. Full frame transfer (FFT) devices are optimised for low noise operation by slowing the scan rate. Figure 35 compares common CCD architectures.

#### CMOS Imagers

Like CCDs, these imagers are made from silicon. Unlike CCDs, each pixel has its own integrated amplifier. Where CCDs pixels always transfer charge, CMOS pixels converts this to a voltage, enabling faster clocking speeds and hence frame rates. The relative advantages/disadvantages are described in Figure 36 and Figure 37. Since CMOS sensors have readout transistors at every pixel, the active pixel area (fill factor) is reduced. Typical CCD cameras have 5 to 10 times the sensitivity of CMOS cameras, making them more suited for faint/low light conditions. They compensate for this by taking longer exposures, and stacking more frames. Binning is also often used to increases sensitivity. This technique combines the charge from adjacent pixels at the expense of spatial resolution.

#### Multi-port/Multi-gate CCDs

These devices use multiple amplifiers so that parallel readouts can be performed. This significantly improve frame rates, and like CMOS cameras fast frame rates up to 10 KHz can be achieved.

#### Intensified CCD (ICCD)

ICCDs utilise an image intensifier coupled to a CCD. They offer high sensitivity in ultra-low-light-level conditions. Since the intensity is increased, the exposure time can be reduced and gating methods can be utilized to provide better temporal resolution, allowing the capture of transient events. These cameras are also suited for lifetime imaging.

#### Digital APDs/Photon Imagers

Another contender to the ICCD is the digital APD or photon imagers. Si-Photomultiplier technology has been combined with CMOS technology to form a new generation of low light cameras currently under development by SensL [67]. The significant difference between this architecture and traditional CMOS/CCDs is that these devices operate in Geiger mode, allowing them to be extremely sensitive to individual photons of light. These systems will have the capability to photon count at the pixel level, making them very sensitive. This could allow for exposure times to be reduced even further, allowing the capture at finer timescales.

#### Time delay integration (TDI)

This is an effective method for imaging moving objects. Normally, an image is detected as a signal charge of each pixel. The image must stay fixed during the charge integration time. If an object is moving, the image can become smeary. The TDI's CCD has rows of pixels with charge transfers that are synchronized with the speed of the moving object. This technique allows clear imaging of objects moving at line rates up to 100kHz [63]. Future improvements of this technique may prove useful for applications such as high speed turbine blade imaging.

#### Noise

Noise is unwanted signal that prevents accurate measurements and evaluation. The main contributors are summarised in Figure 38.

## 10. Excitation Sources

The energy for excitation can be supplied to luminescent molecules by a variety of ways. Examples include electromagnetic radiation (lasers and LEDs), particle beams (electrons, neutron, ions) and electrical current. This section only reviews electromagnetic radiation, focusing mainly on UV lasers.

### 10.1 Pulsed Laser Systems

#### Nd:YAG Laser Systems

The advantage of temporal approaches, such as the lifetime decay analysis, is that it is independent of illumination intensity, phosphor concentrations and thickness, and therefore less prone to errors common in the conventional intensity method. The disadvantage of this method is that it lacks signal strength, since the excitation light is only available for only fractions of the time.

From the literature, it seems that most researchers have used nitrogen lasers (337 nm) or Nd:YAG lasers in the third (355 nm) or fourth harmonics (266 nm) to produce high energy pulsed UV light. These lasers have been an excellent choice for phosphor illumination. The state of this technology has advanced in the past few decades, and present Q-switched solid state laser system can be expected to deliver around 500 mJ at 355 nm and 200 mJ at 266 nm with repetition rates of around 20 Hz and pulse duration of 10 ns [68, 69]. Other newer technologies include diode pumped solid state (DPSS) Nd:YAG lasers. High powered pulsed laser systems may not be suited for phosphor illumination as fluence (energy/area) may be too great for the phosphor and issues such as sputtering and the breakdown of air may become more relevant at energies >30 mJ. However, high-energy laser beams could easily be expanded to reduce fluence and be used to produce large UV illumination areas suited for lifetime imaging purposes.

#### Q switched diode pumped solid state (DPSS) laser

These lasers have laser diodes, instead of flash lamps, to pump the solid gain medium. They have replaced many flashlamp lasers in many scientific applications. Pumping efficiencies are greater since the diode's narrow wavelength is usually optimised for peak absorption. Flash lamps generate broader wavelengths, with additional light that is not adsorbed. Typical high energy systems deliver 1 mJ per pulse in the UV range. This is relatively lower than flashlamp-pumped alternatives. NASA is currently developing the state of the art DPSS laser system with the goal of transmitting pulse energies greater than 200 mJ in the UV range [70]. DPSS lasers offer higher repetition rates (500 to 5,000 Hz). Lot-Oriel Group [71] have produced a DPSS Nd:YAG laser capable of delivering 250 mJ/pulse at 1,064 nm with a repetition rate of 400 Hz and a pulse width of 800 ps. Assuming a typical reduction in power by an order of magnitude to frequency triple, or quadruple the laser beam to produce UV wavelengths, this equates to approximately 25 mJ/pulse.

#### Excimer Lasers

These are gas lasers formed by a mixture of three different gases: a rare earth gas (e.g. Ar, Kr, Xe), a halogen (either F or Cl), and a bath gas (Ne or He). An advantage of excimer lasers is that they produce high power pulse outputs directly in UV range, and no frequency tripling/quadrupling is required, that typically reduces the energy by an order of magnitude. Typical high energy excimer lasers produce 200 mJ energy/pulse at wavelengths ranging from 157-351 nm [72]. They typically operate with at a repetition rate of around 500-1000 Hz, with pulse duration near 10 ns. More powerful models, such as Coherent SX series, offer an impressive 1000 mJ/pulse at 300 Hz [73]. According to Junger and Schmidt [74], excimer lasers still remain unchallenged as the only source laser to deliver high pulse energies and high average powers at UV wavelengths.

In the past, excimer lasers had issues with working lifetimes, laser pulse stabilities and performance. However, they have vastly advanced in the past few decades with increased gas, tube life and pulse homogeneity [74]. Pulse stabilities have improved from ±12% to ±2%, with a jitter of less than 2ns. This performance is still slightly lower than Nd:YAG systems with typically stabilities of 1% and jitter of 0.5 ns. Recent improvements in Junger and Schmidt [74] have reported energy stabilities to have improved to ±0.5%.

Excimer lasers produce quasi-rectangular beams, typically 8×20 mm, with a near-Gaussian profile in the short axis, and a super-Gaussian profile in the long axis (Figure 39). Due to the shape and intensity profile, transformations cannot be made using the same optical systems used for round Gaussian beams. Most applications require the beam to be modified by homogenizing and then reshaping the profile to match the application. This can add complexity in the optical system.

### 10.2 Continuous lasers/light sources

Continuous lasers and other light sources are suitable for intensity measurements. Pulsing can be introduced to enable lifetime mode analysis. One way is to use a mechanical shuttering mechanism. There are limits on how fast these can operate. Previous mechanisms operated in the sub milliseconds regime, and were considered too slow and unsuitable to detect lifetimes shorter than this. Newer optical choppers/mechanical shutters can provide sub-microsecond [75] pulses, and now there are a range of optical shutters (LCDs) that can provide responses faster than this. The problem with continuous lasers is that the energy per pulse is relatively low. For example, to obtain the same power as you would from a typical 266 nm 10 ns 10 mJ Q switched Nd:YAG laser pulse, you will need a continuous laser operating continually with 1 MW of power at that wavelength. Typical high energy laser systems only operate with average powers of 5 KW. This lack of power from continuous sources have lead to researchers resorting to high peak power pulsed laser systems, even when they are utilising the intensity mode [29, 33, 76].

### 10.3 Fibre Lasers

Fibre lasers are increasing becoming more popular due to increased reliable up-time, beam quality, reduced running costs and servicing operation. In principal, fibre lasers are similar to DPSS lasers. The generic design includes laser diodes for pumping; a scheme for coupling the pump energy into the gain medium; a fibre based resonator configuration with brag gratings instead of mirrors; and a method for getting rid of excess heat. In a fibre laser, the laser is created directly inside a fibre. Therefore, there is no need for optical setup that requires the beam to delivered to a target via a series of steering mirrors. The use of fibre optics opens up areas of application that may have restricted optical access. Fibre lasers eliminate the need to for fibre optic coupling from a conventional laser. Fianium Ltd has recently created the worlds' first commercial high-powered 266 and 355 nm UV fibre laser [77]. The system is capable of producing 1W average power at 100 MHz, with a pulse width of 10 ps. This yields to 0.01 uJ/pulse and a peak power of 1 KW, which is relatively low.

### 10.4 UV LEDS

Allison *et al.* [78] reports that high energy UV LEDs could be used to excite phosphors. They can be used in continuous or pulsed mode. In the pulsed mode, even though they have relatively low powers, they can be operated with pulse widths to increase the total amount of pulse energy. They have fall times of a few ns, and have emissions spectra much broader than that of a laser. This may be absorbed better by the phosphor, and may produce intensities that are of similar magnitude. Newly developed high powered UV LEDs can produce 450 mW of continuous 380 nm UV light [79]. In pulsed mode at a pulse width of 1 µs yields to energies of around 0.45 µJ/pulse. This is relatively low and would therefore be unsuitable for high temperature measurements where blackbody radiation becomes significant. It may be suitable in applications where relatively low temperatures are concerned.

LEDs have been very successful and have replaced lasers in many applications including pressure-sensitive-paints and fluorescence detection for biological purposes [80]. Since the pulse widths can be finely controlled, the decay lifetime can be determined using the frequency domain approach highlighted in Section 5.2. A sinusoidal wave using blue LEDs was used to excite the phosphor and determine lifetime in experiments conduced by Allison *et al.* [42].

## 11. Survey of recent applications using thermographic phosphor

The use of thermographic phosphors to determine temperature has been successful for a number of applications. This section briefly surveys some that have been reported in the past few years.

### 11.1 Impinging Jet Flame Experiment

**Figure 12.**
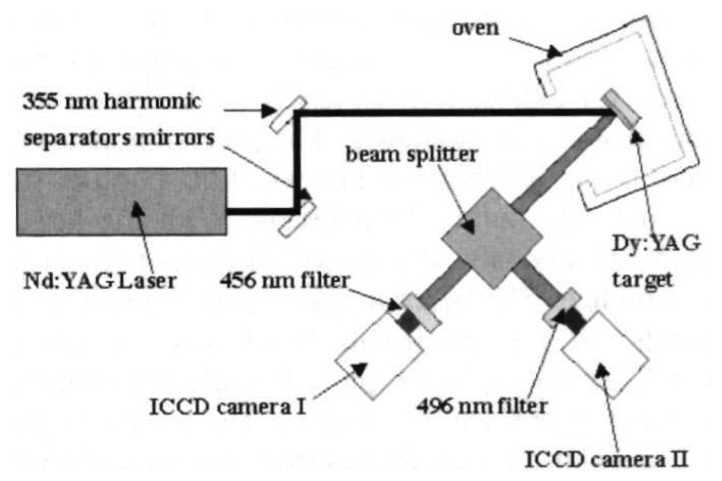
Schematic of the intensity ratio thermal imaging system [32].

**Figure 13.**
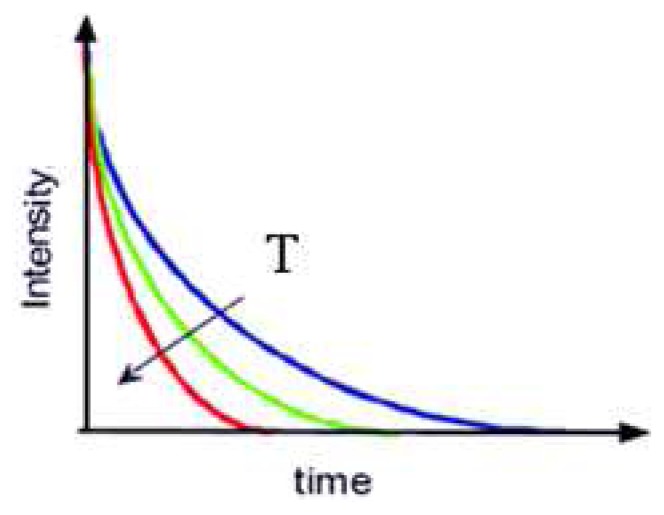
Typical lifetime characteristics with increasing temperature.

**Figure 14.**
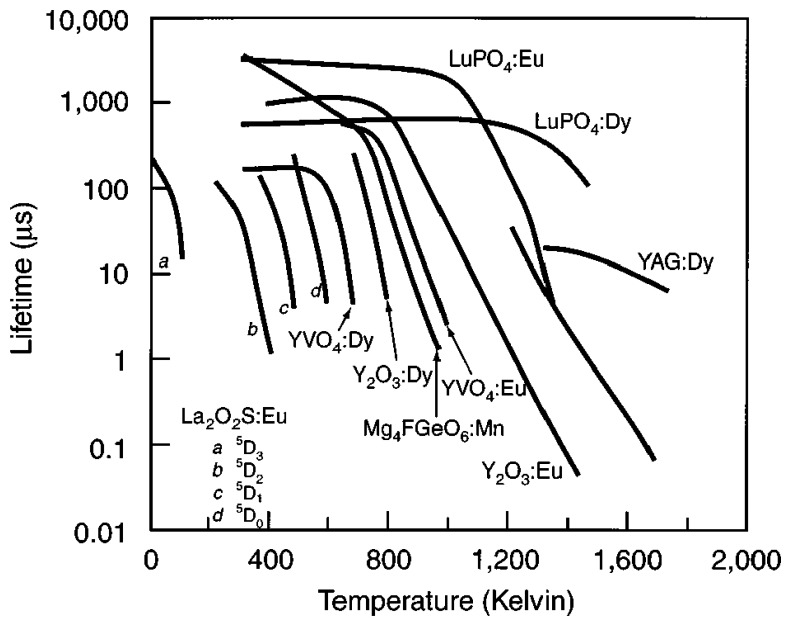
Lifetime of phosphors vs. temperatures. Taken from Allison and Gillies [2].

**Figure 15.**
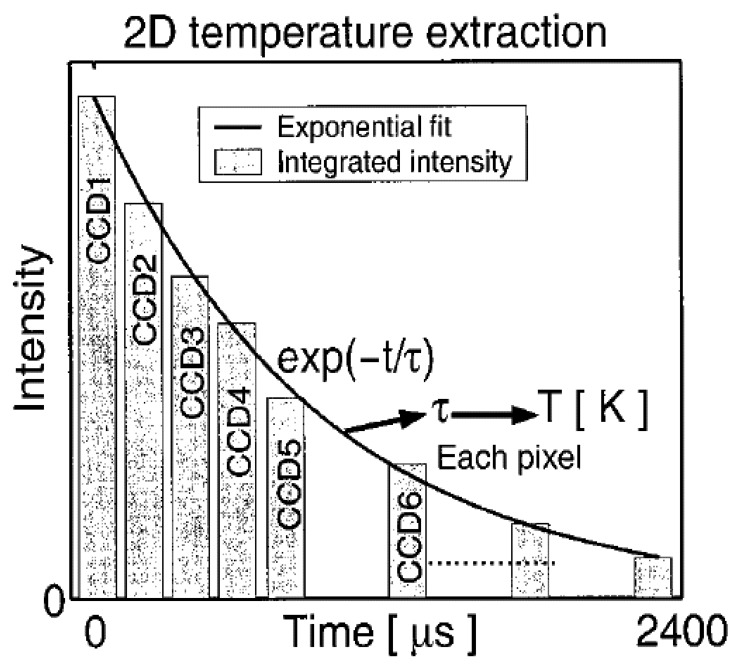
Curve fit for a single pixel from a series of images obtained from 8 CCD detectors [40].

**Figure 16.**
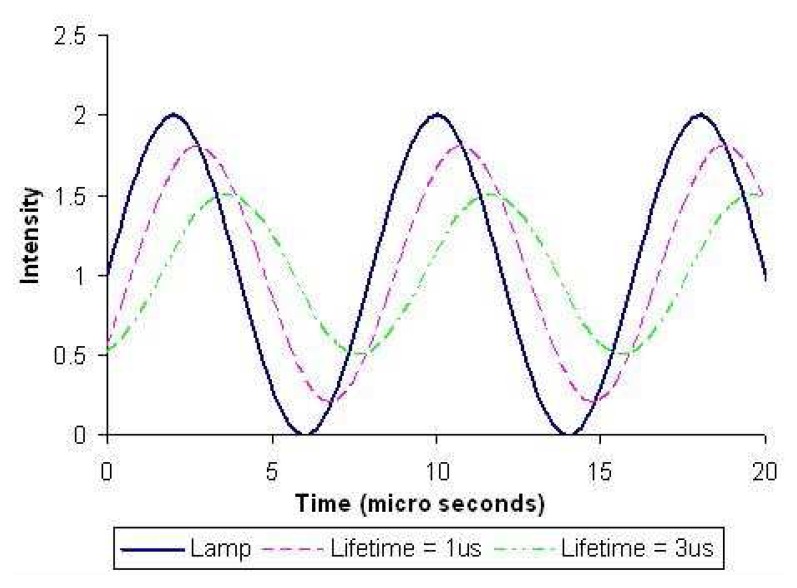
Phase shifts for different lifetimes.

**Figure 17.**
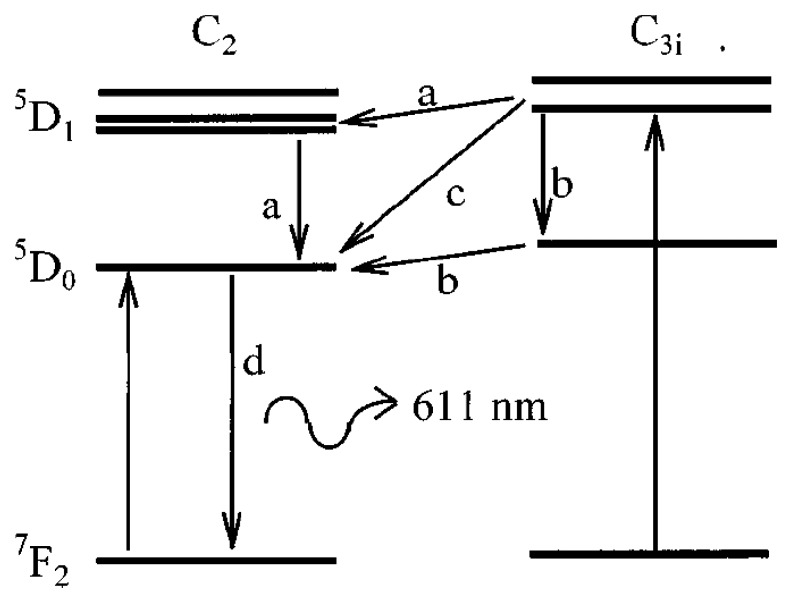
Energy levels of Y_2_O_3_:Eu at symmetry sites C_2_ ad C_3i._

**Figure 18.**
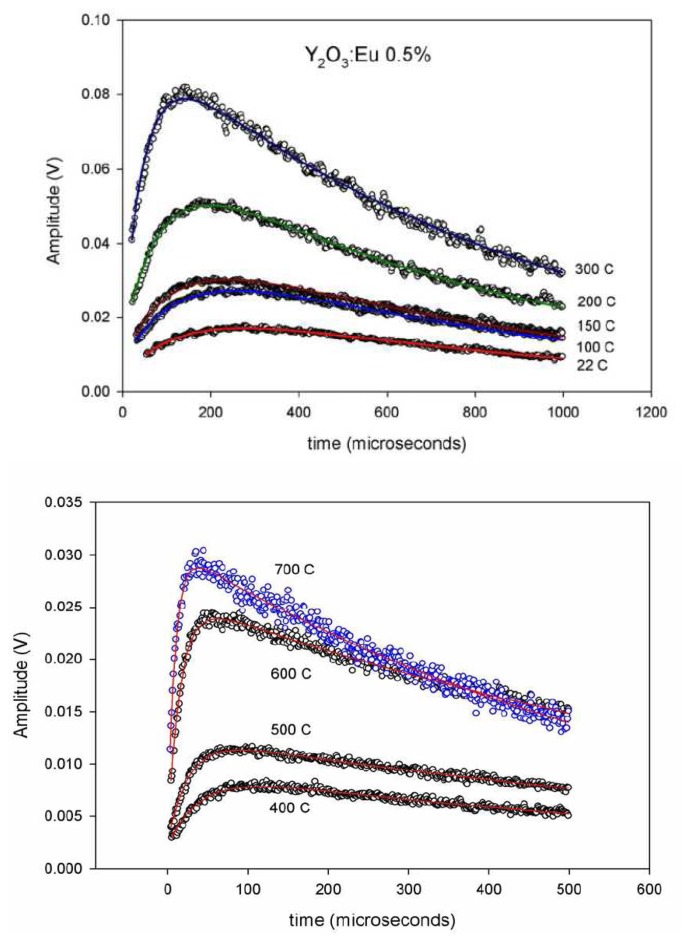

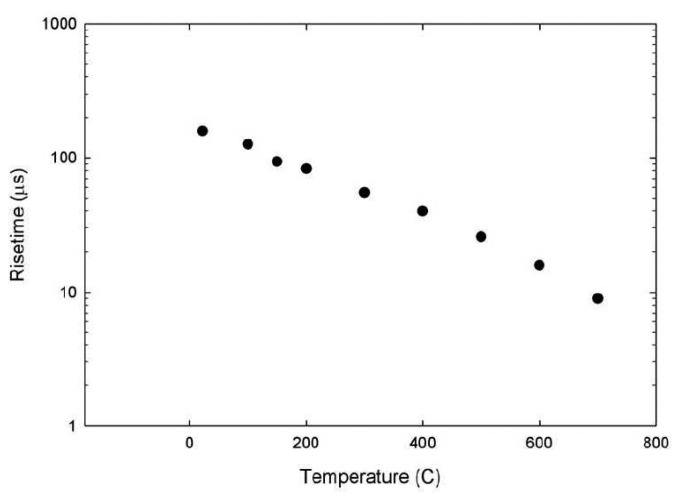
Risetime variation with temperature. Taken from Allison *et al.* [48].

**Figure 20.**
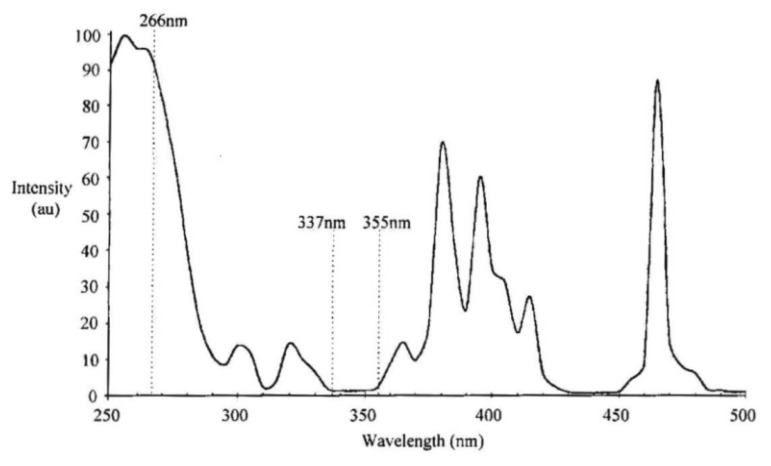
The absorbtion spectra of Y_2_O_3_:Eu [20].

**Figure 21.**
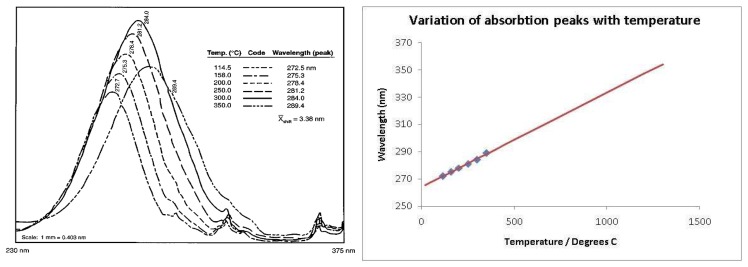
Left: Variation of absorption peak with temperature [2]. Right: Trend line predicting peak absorption wavelength at higher temperatures.

**Figure 22.**
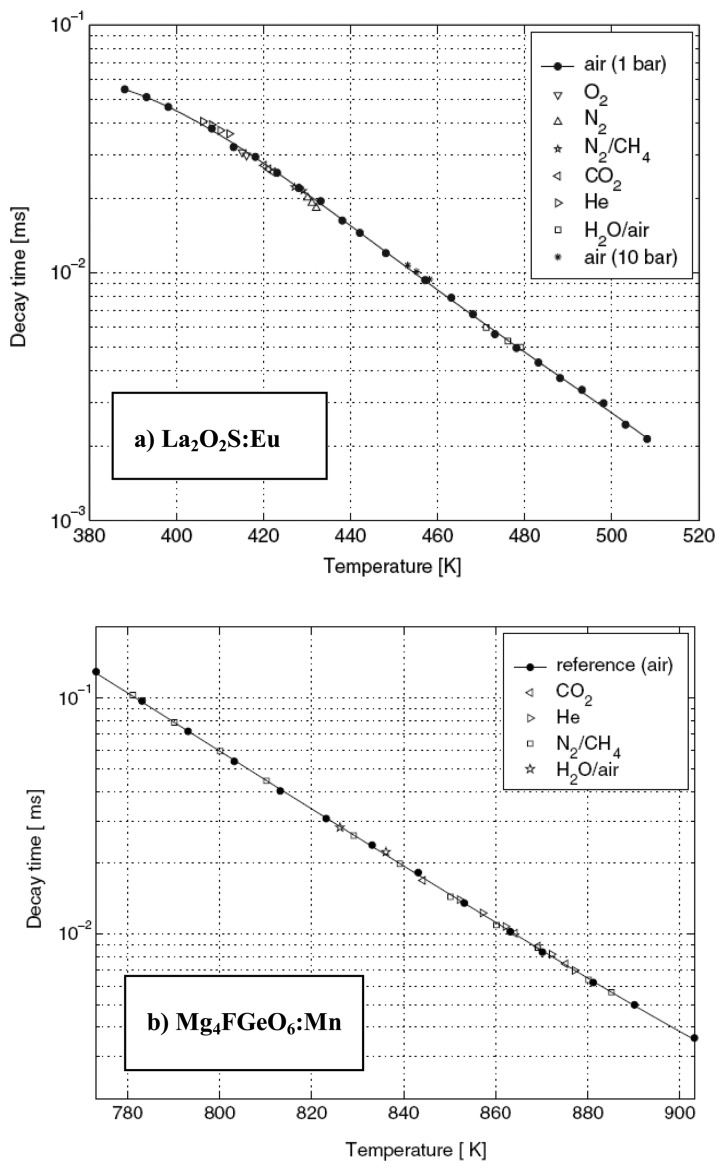

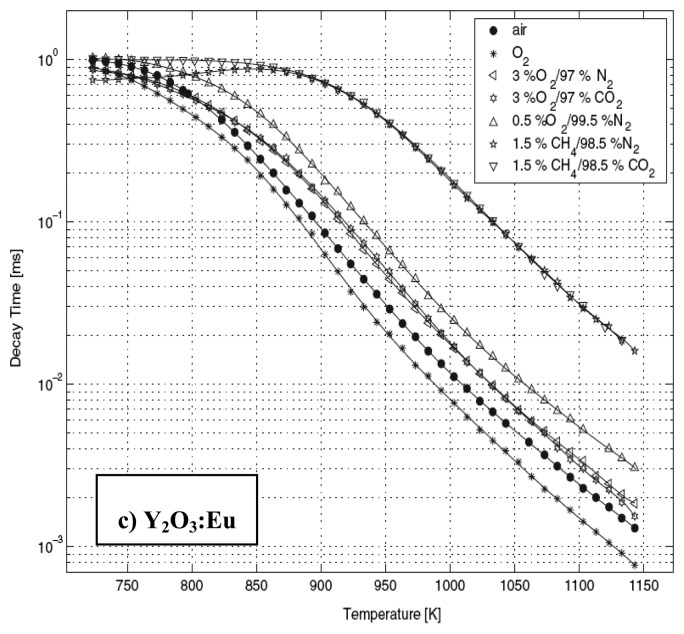
Effects of different gases on the lifetime decay of different phosphors at different temperatures. Phosphors a) La_2_O_2_S:Eu, b) Mg_4_FGeO_6_:Mn, c) Y_2_O_3_:Eu [52].

**Figure 23.**
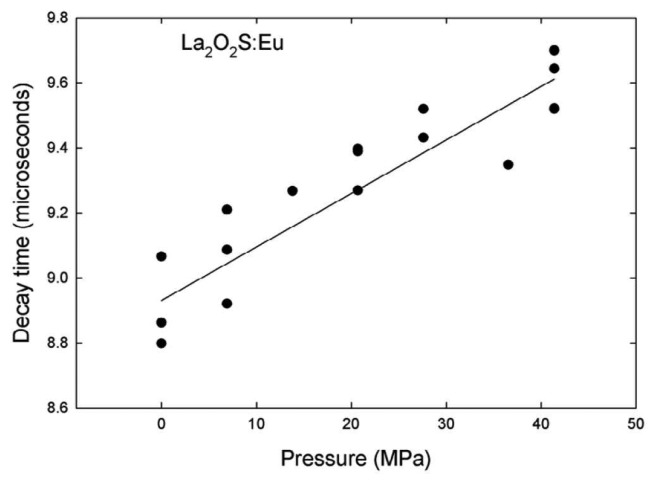
Variation in lifetime decay time of La_2_O_2_S:Eu phosphor with increasing pressure [53].

**Figure 24.**
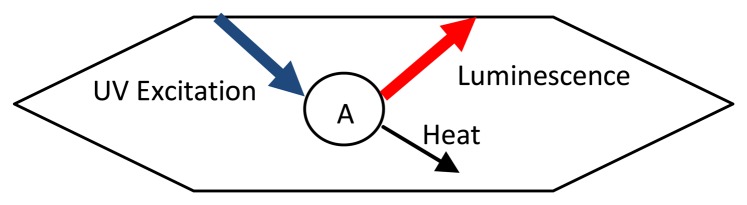
UV Excitation energy acting directly on the activator.

**Figure 25.**
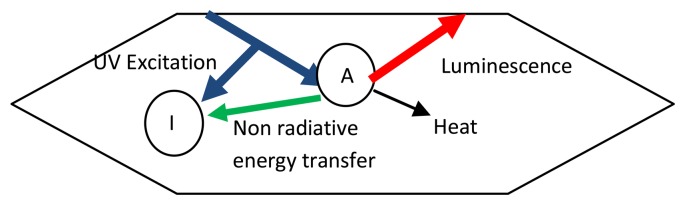
Interactions between excitation energy, impurities and activator.

**Figure 26.**
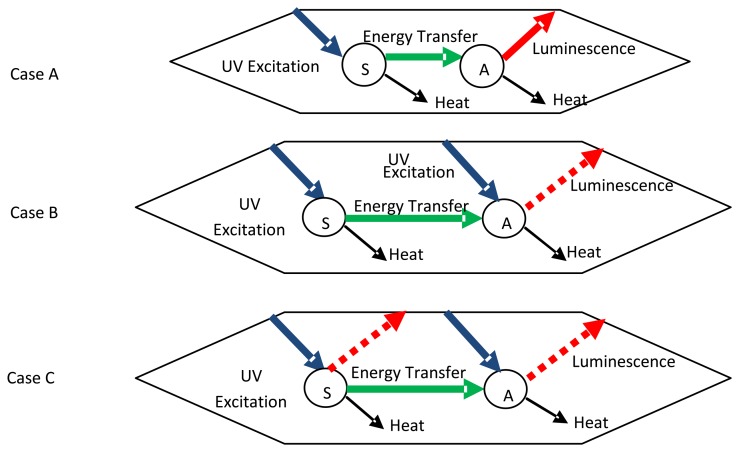
Examples of interactions between excitation energy, sensitizer and the activator.

**Figure 27.**
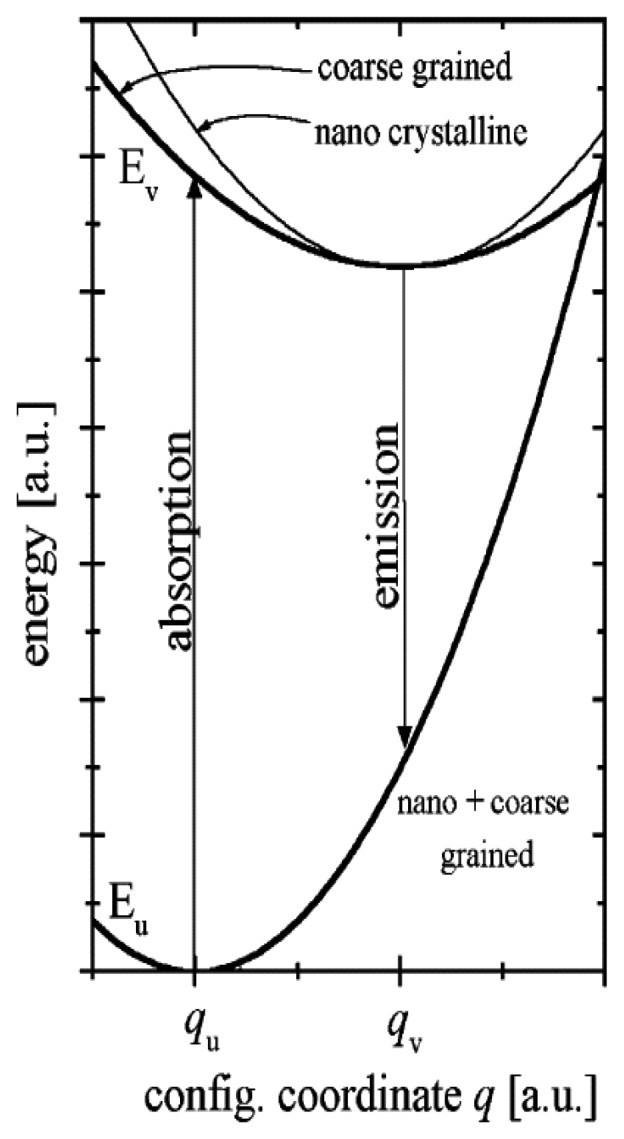
Effects of reducing the particle size [54].

**Figure 28.**
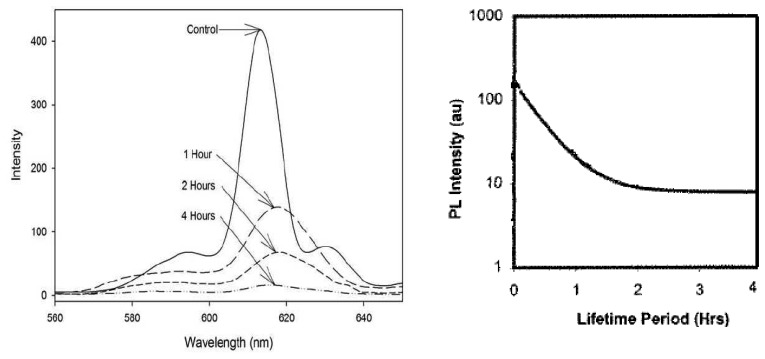
Left: emission spectra of Y_2_O_3_ phosphor in Resbond binder after thermal exposure to 1,400°C [59]. Right: Intensity of the peak emission of Y_2_O_3_ phosphor after thermal exposure to 1,200°C [60].

**Figure 30.**
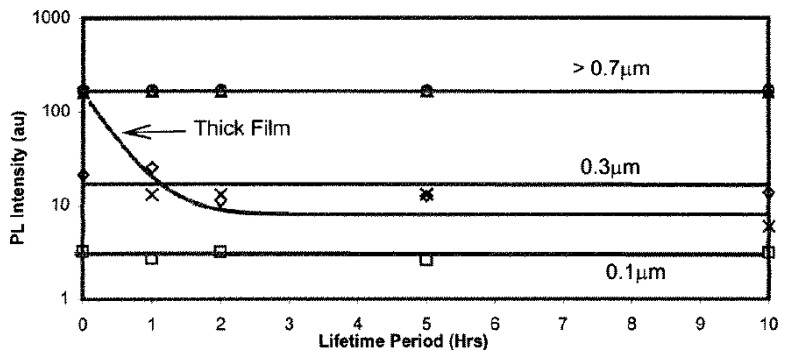
The variation in intensity with time (hours) at a constant thermal exposure of 1,200ºC for thin coatings (0.1 µm-3 µm) and thick film coatings (10 µm) [60].

**Figure 31.**
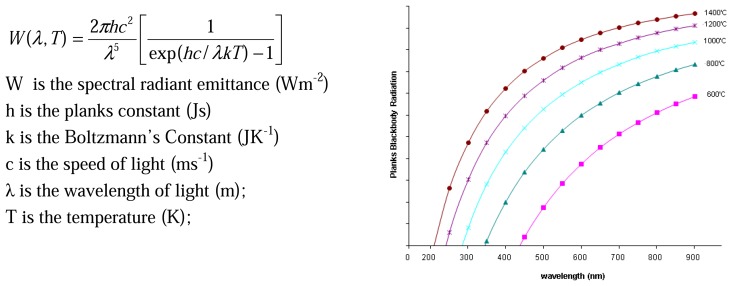
Distribution of blackbody radiation at different temperatures.

**Figure 32.**
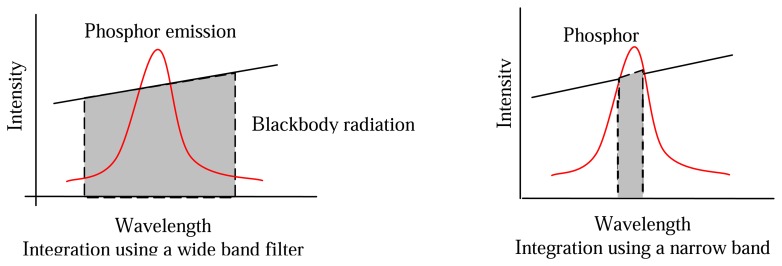
Figure to show the amount of background radiation (shaded area) collected using wide and narrow band filters.

**Figure 33.**
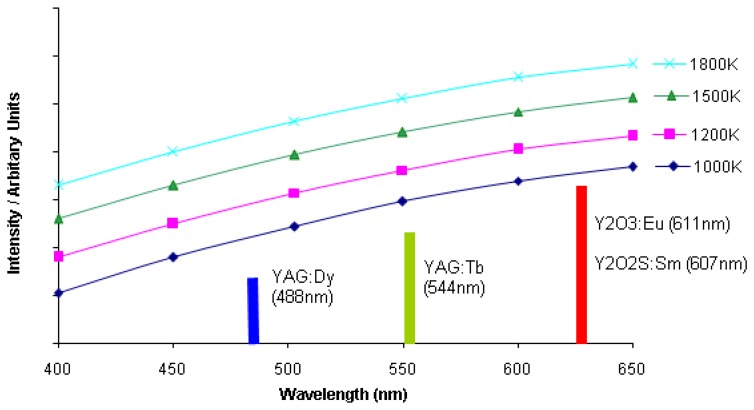
Phosphor intensity (at their peak emission wavelengths) required to maintain similar signal to blackbody radiation.

**Figure 34.**
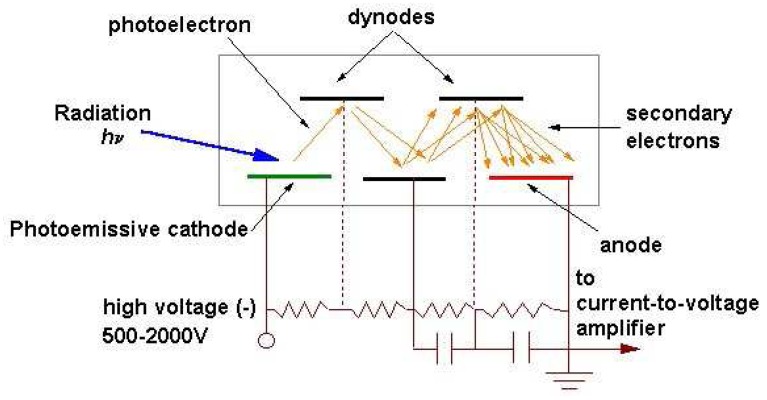
Schematic of a PMT.

**Figure 35.**
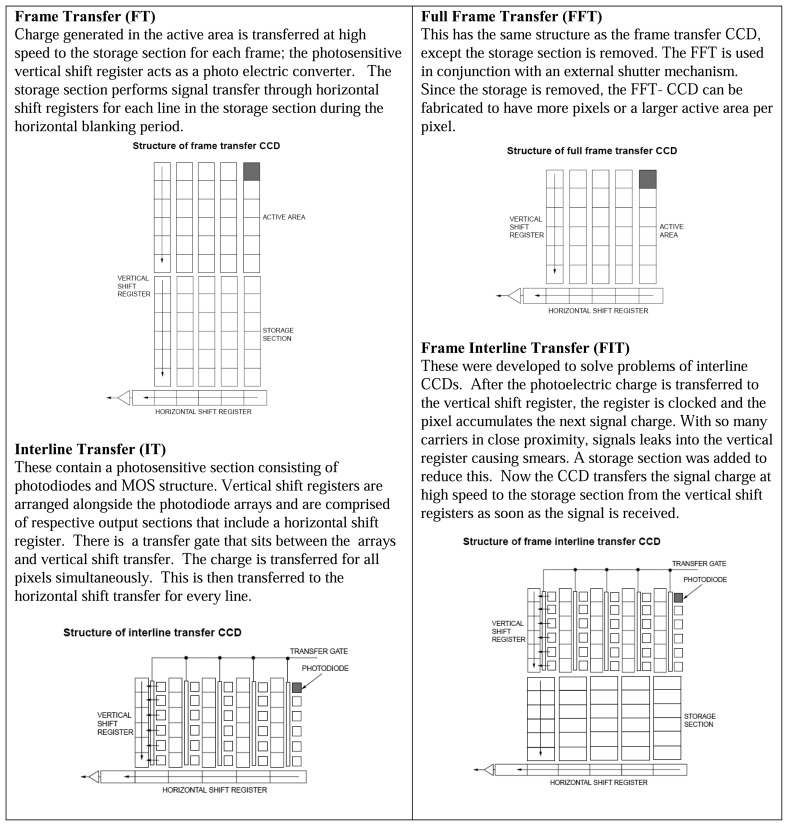
Comparison of different CCD cameras. Pictures taken from Hamamatsu [63].

**Figure 36.**
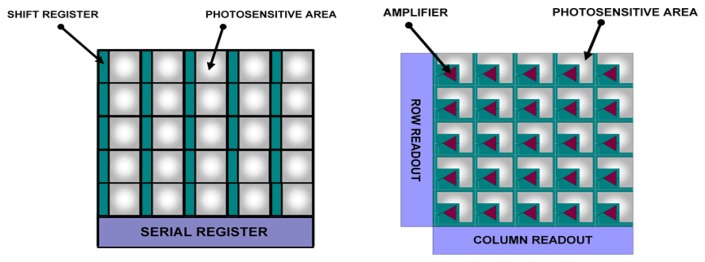
Comparison of CCD (left) and CMOS (right) cameras.

**Figure 37.**
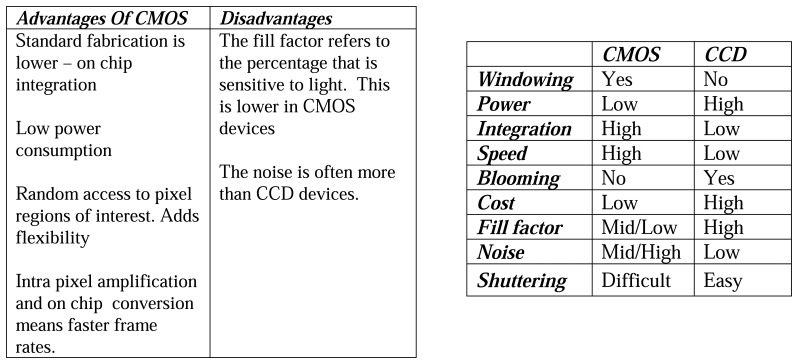
Comparison of CCD and CMOS cameras.

**Figure 38.**
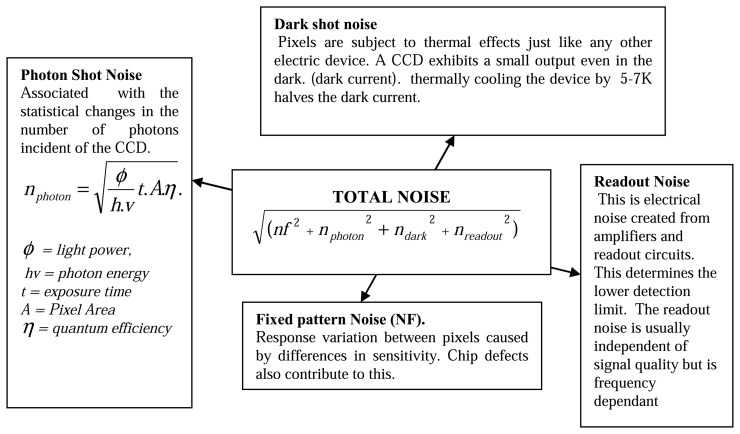
Noise from CCDs.

**Figure 39.**
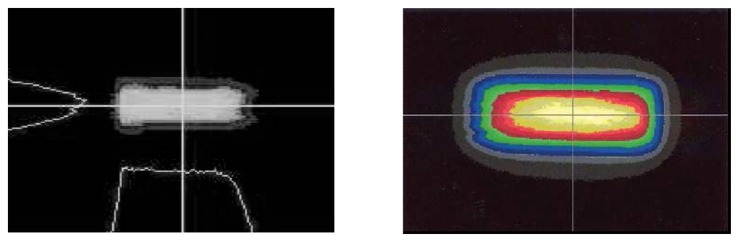
Typical rectangular excimer laser profile.

Kontis *et al.* [32] investigated a turbulent flame that was impinged onto a 100 µm YAG:Dy phosphor coated onto an alumina ceramic disc of thickness 0.05 m and diameter of 0.035 m. The intensity ratio method was utilised to map the temperature every 2.5 s for a total of 142.5 s for heating, and 150 s for the cooling of the plate. The system used was previously shown in Figure 12. The results show that the maximum temperature (1250K) was attained after 135 s of initial flame impingement. The temporal variations in the temperature profiles around the centre of the disc is an indication of the strong and localised effects of hot jets, and the transient heat over the entire disc surface. Kontis *et al.* [32] notes that such systems can be used to evaluate the local heat transfer coefficients by using an appropriate theoretical model of the thermal response to heat flux of the configuration under consideration.

Similar transient heating experiments using the intensity ratio method was also undertaken by Heyes *et al.* [34]. A ceramic plate was brush-painted with YAG:Dy forming a thickness of 150-200 µm. A Nimonic alloy was coated with YSZ:Dy using plasma spray, producing a thickness of 100 µm. A Bunsen burner was ignited after 20 s, and was used to heat the rear surface the plates for 60 s. A K-type thermocouple was mounted on the front surface for comparative analysis. A Nd:YAG laser, with a pulse energy of 50 mJ, was used to excite the phosphors. Ten pulses were averaged for each measurement with a sampling period of 1 s.

**Figure 42.**
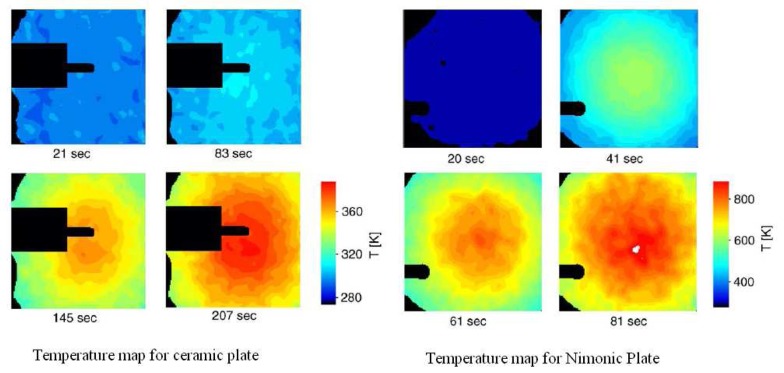
2D temperature map for ceramic and nimonic plate at different times. Taken from Heyes *et al.* [34].

**Figure 43.**
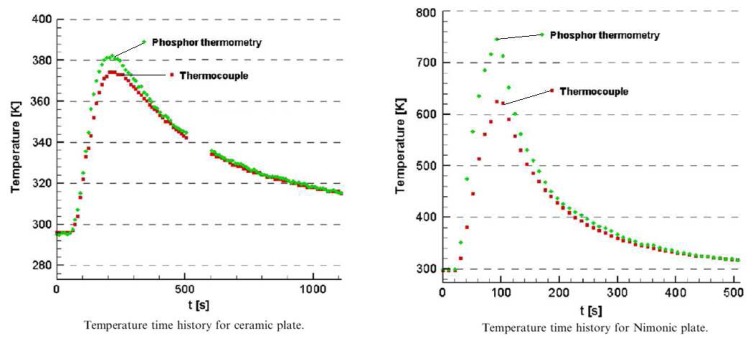
Temperature history for ceramic and nimonic plates. Taken from Heyes *et al.* [34].

Results, shown in Figure 42 and Figure 43, for the ceramic plate heating indicate that the front surface of the plate continued to heat up after the heat was removed. This is to be expected from ceramic material that has low thermal conductivity. The maximum temperature recorded was at 390K at 207 s. Results for the Nimonic plate indicate that the maximum temperature was observed at 840K after 82 s. Thermocouple measurements were in good agreement below 400K (5K difference). At higher temperatures there were larger differences, highlighting the difficulty of accurately measuring temperature of hot surfaces using conventional methods. Heyes *et al.* [34] notes that thermocouples are expected to under-predict the temperature due to changes in the local temperature caused by thermocouples acting as a heat sink, imperfect contact, heat loss by thermocouple convention and radiation, with additional errors caused by the thermocouple junction being above the surface.

### 11.2 After burner experiments

**Figure 44.**
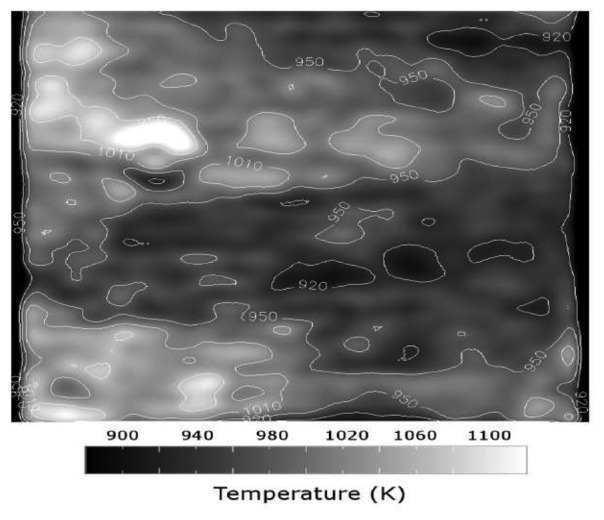
Surface temperature map of a section inside an afterburner [82].

Phosphor thermometry to measure the temperatures of surfaces within operating turbine engines dates back at least 20 years. Noel *et al.* [81] proposed such system in 1986. In a study by Saner *et al.* [82], optical temperature diagnostics was performed on the afterburner of an Volvo RM12, the engine used in the Gripen fighterjet. The intensity ratio mode of YAG:Dy was used to map the temperature inside an afterburner. HPC binder paint was used to attach the phosphor on to the surface of interest. A 355nm Nd:YAG laser was used to excite approx 100 mm^2^. The phosphorescence signal was sampled using a stereoscope and an ICCD, allowing the simultaneous imaging of two spectrally filtered images. The ICCD was gated at 100 µm to suppress background radiation. The images of 455 nm and 493 nm were digital divided to obtain a ratio at each pixel that was processed through a calibration curve to reveal temperature. Temperature maps were taken at different engine running conditions. Figure 44 shows thermal maps at full afterburner load.

The measurements corresponded well to previous measurements made with thermocouples. This experiment demonstrates phosphor thermometry could be used in very harsh environments. In addition to increasing blackbody radiation at high temperatures, another problems that was encountered was that of flame emissions that overlapped emissions from the YAG:Dy phosphor. The flame emissions peaked at approx 415 nm. Although, background images were subtracted, it would have been more beneficial to use phosphors with emission wavelengths that did not interfere with the environment. In more recent experiments involving afterburner temperatures, the lifetime approach was used to determine temperature using an 266 nm Nd:YAG laser, a PMT and Mg_3_FGeO_4_:Mn phosphor [83].

### 11.3 Combustor Rig and Film Cooling

Surface temperature measurements were made in a laboratory combustion rig by researchers at Imperial College, London [22]. The system was used to assess whether thermographic phosphors could be used for surface temperature measurements in gas turbines, and to evaluate film cooling. Film cooling involves the ejection of air over a surface, and is generally used to cool combustor liners, turbine blades and nozzle guide vanes in gas turbines. It provides thermal protection from high gas temperatures that usually exceeds the materials melting point, and would otherwise cause failure.

**Figure 45.**
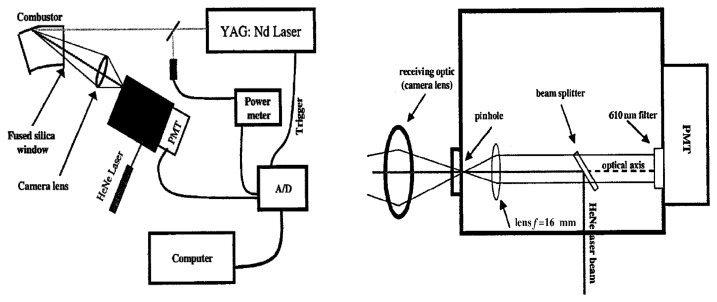
Optical arrangement for combustor measurement [22].

The combustor rig had a quartz window for optical access. Cooling air was directed over the window by a row of holes to enable it to survive the temperature, while keeping it free from wetting and carbon build up. The system was first tested using 20 µm Y_2_O_3_:Eu paint, prepared by Roll-Royce Plc, over an underlying TBC of thickness 250 µm. The phosphor was also applied within the depression of the cooling hole exits. The optical setup was arranged so the lifetime decay response could be measured using a PMT (Figure 45). A camera lens was used to focus the detection on a single point, and a traverse was used to successively scan an area of 8mm^2^ with 128 point measurements.

This system evolved and was later modified to simultaneously measure both the intensity ratio and the decay response of YAG:Dy phosphor, allowing temperature to be determined by two independent methods [84]. A dichroic mirror was used to split the light equally to two PMTs that had spectrally different filters capture the relevant wavelengths of 455 nm and 494 nm. A schematic is shown in Figure 48.

**Figure 47.**
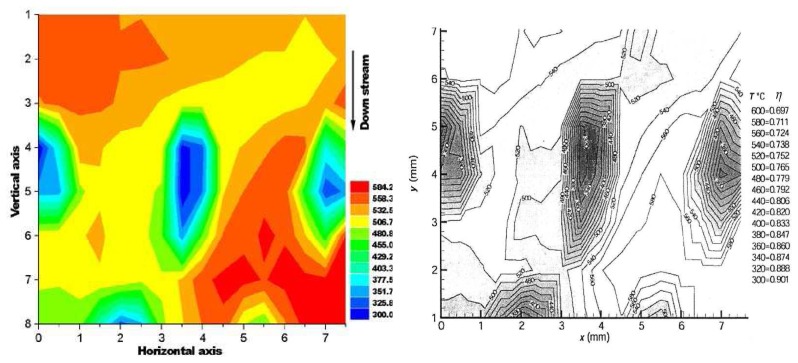
Results showing the temperature distribution/cooling effectiveness inside the combustor: (left)Contour map [22]; (Right)Colour thermal map [84].

The results (Figure 47) indicate a temperature distribution ranging from 300–600°C, with the uncertainty reported to be better than 2% [22]. The effects of film cooling holes can be clearly seen, demonstrating phosphor thermometry's capability in measuring such phenomena.

Both efficiency (fuel economy) and performance (thrust) can be improved without increasing the size of the engine if higher turbine inlet temperatures are achieved [85]. However, there are consequences; a few degrees of operation at over-temperature can result in drastic reductions in blade life. Therefore, the operating temperature is prescribed by the balance between the benefits of thermal efficiency at higher temperatures and material stability and life.

At the moment, Khalid and Kontis at the University of Manchester are researching into methods of successfully measuring surface temperatures on both rotating and static components of development aeroengines using phosphor thermometry. This will eventually help predict heat transfer distributions, verify the effects of design changes, cooling effectiveness, and aid designers optimise aeroengine designs to enable higher temperature operation.

### 11.4 2D surface - Thermal lifetime imaging

Lifetime imaging for thermal measurements using thermographic phosphors has been intensively used by a team of researchers at Lund University. They claim to be the first to obtain such 2D measurements [40]. A high speed framing camera (Imacon-Hadland), containing an eight faced prism is used to split light to eight independent intensified CCD cameras, where the exposure time and time separation between images can all be precisely configured.

**Figure 48.**
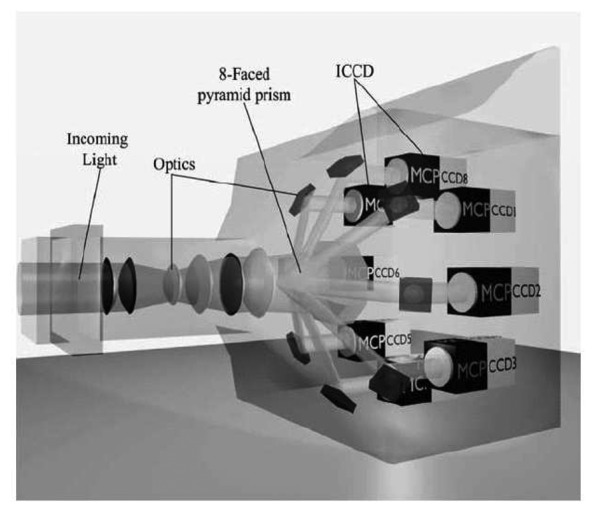
Architecture of the framing camera, showing the eight faced prism splitting the light equally to eight ICCD cameras [86].

**Figure 49.**
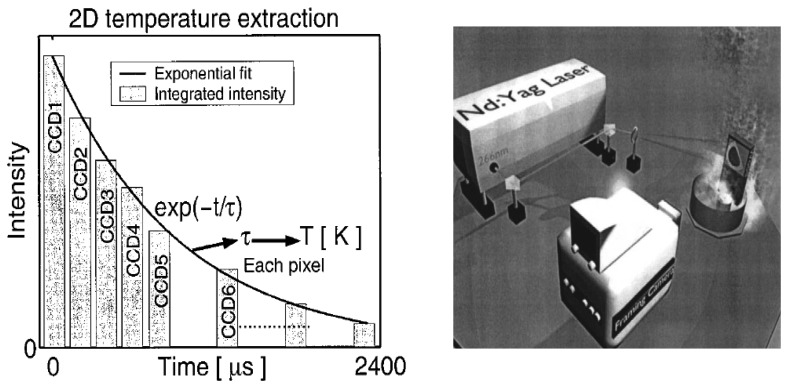
Left: Curve fit for each pixel from intensities integrated from 8 CCD detectors; Right: Schematic for temperature measurement. Pictures taken from [40].

Figure 48 shows a schematic of the framing camera. Using these images, an exponential curve can be fitted for each pixel, and evaluated against a calibration curve (Figure 49). This produces a complete 2D thermal map that was determined using lifetime decay analysis.

The group have obtained 2D surface measurements on low density fibre boards covered with Mg_4_FGeO_6_:Mn phosphor. Burning alcohol was used to heat the sample because it generates less soot that could interfere with measurements.

**Figure 50.**
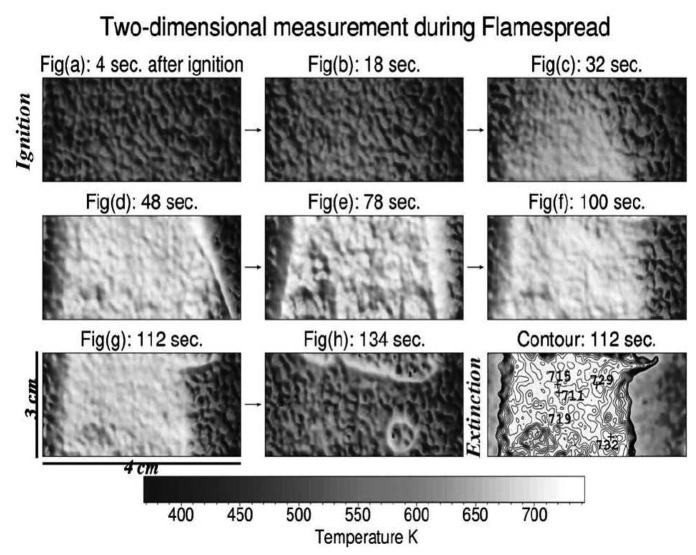
Temperature measurements during flame spread. 2D surface temperature measurement of low density fibreboard (LDF) [86].

The radiation energy was 24 mJ/pulse which was expanded to an area of 100 cm^2^ yielding a fluence of 0.25 mJ/cm^2^. The results obtained showed a standard deviation of ±5K (less than 1%) at temperatures between 680-780K [40]. Recent experiments involved measurement of combustible and non-combustible surfaces using both the intensity ratio and lifetime imaging modes [86]. Figure 50 shows thermal temperature maps during a flame spread experiment at various times.

### 11.5 Surface Temperature Measurements of decomposing materials

The surface temperature is a key parameter for modelling the decomposition of solid materials. It has a strong influence on the heat flow into and out of the material, and also determines the ignition temperature. Omrane *et al.* [87] used phosphor thermometry for surface temperature measurements in pyrolysis studies. Pyrolysis is the chemical decomposition of organic materials by heating in the absence of oxygen or any other reagents. According to the authors, accurate measurement using conventional methods prove to be problematic. Thermocouples and thermistors require direct contact, and may suffer heat losses and induce catalytic effects. Pyromerty can be difficult because emissivity of the surface may not be known and may be changing during the investigation.

**Figure 51.**
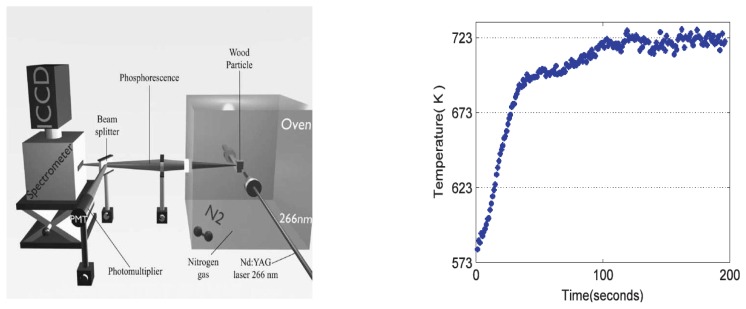
Schematic and results from the pyrolysis study for birchwood [40].

Phosphor particles were deposited on the material under investigation and were placed inside a high temperature reactor that was pre-stabilised at a temperature of 733K [40]. A 266 nm Nd:YAG laser was used to excite the sample with subsequent emissions collected by a PMT, ICCD and a spectrograph, for both temporal and spectral analysis. A schematic is shown in Figure 51. The results show that the surface temperature increased to reach the temperature of the reactor.

In later work, pyrolysis on construction materials were studied. These included low-density fibre boards (LFB), medium fibre boards (MDF), particle board (PB) and polymethylmetharcrylate (PMMA) [87]. Cubic samples (5 mm^3^) were doped into Mg_4_FGeO_6_:Mn phosphor that was dispersed in ethanol or toluene. The samples were dried at 95°C and inserted through an air cooled fall tube onto to a holder. This was connected to a balance to monitor weight of during the investigation. A molecular beam mass spectrometer was used to monitor gas composition and ensure oxygen levels stayed below 0.1%.

The rapid pyrolysis of construction materials was successfully monitored using phosphor thermometry. The results covered a temperature range of 300-600°C. This covers pyrolysis initiation and completion for most construction materials during typical fire spread situations. The results are another demonstration of phosphor thermometry for detailed temperature measurements during these complex combustion-related situations where common techniques fail.

### 11.6 Internal Combustion Engine valve/piston temperature measurement

Temperature measurement of internal component surfaces enhances understanding of the processes inside the combustion engine. Armfield *et al.* [88], cited in Allison *et al.* [53], reports the use of thermographic phosphors for temperature measurements of an intake value and a piston.

**Figure 53.**
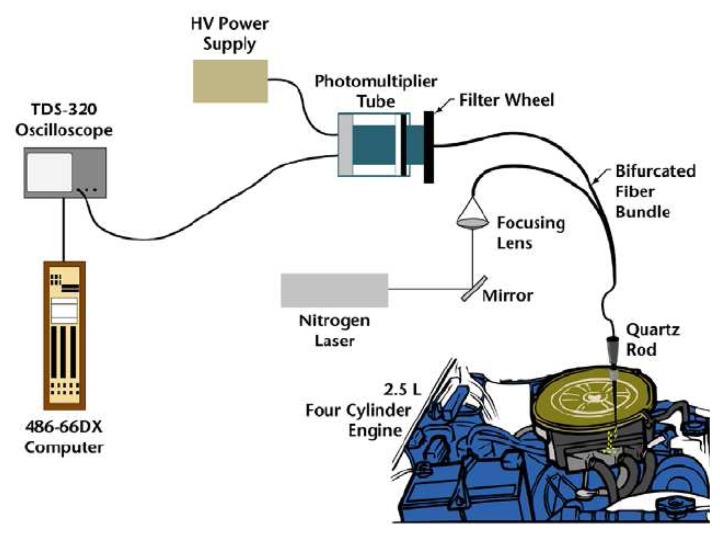
Schematic for intake value measurements.

**Figure 54.**
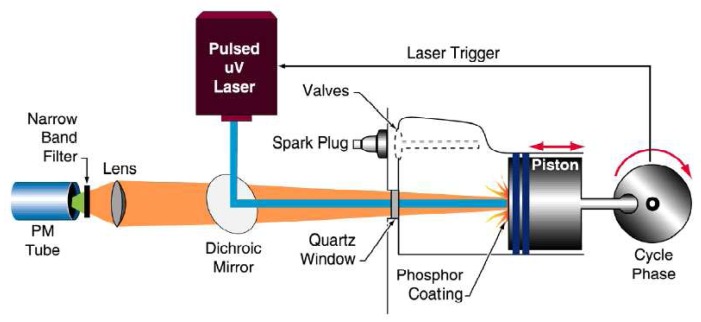
Schematic for piston measurements [53].

For the intake value experiments, LaO_2_S:Eu phosphor was coated on the stem-side of the value. A nitrogen laser was delivered through a 1mm optical fibre accessed through the head of the car. The same fibre was used to direct the luminescent emissions to a PMT. The lifetime decay response was used to determine temperature. A schematic is shown in Figure 53. For piston measurements, a quartz window was used to gain direct optical access as shown in Figure 54.

**Figure 55.**
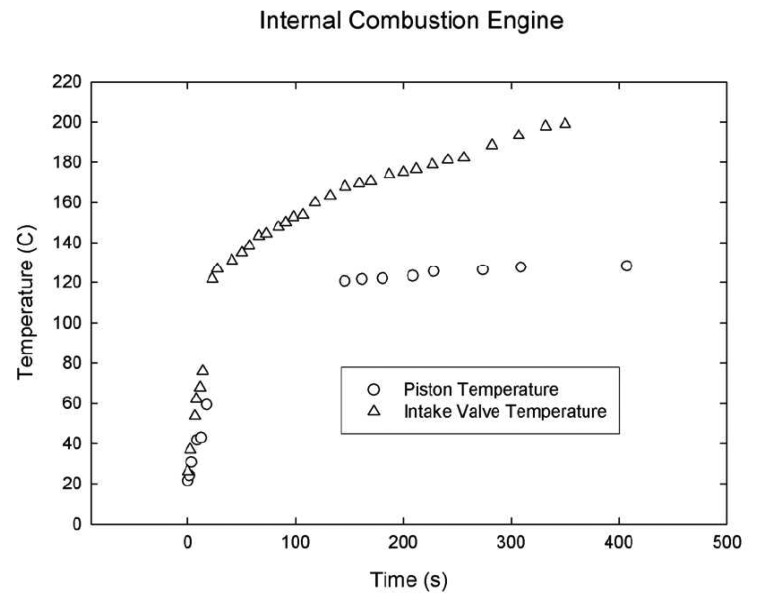
Results for piston and intake value experiments [53].

The results shown in Figure 55 indicate show that in both cases, there is a rapid increase in temperature and is followed by a slower steady increase.

Similar temperature measurements of the intake and exhaust values of an optically accessible laboratory engine was conducted by Omrane [86]. Measurement would increase the understanding of fuel evaporation process when it is in contact with high temperature surfaces. In the investigation, the engine was operated at 1,200 RPM using gasoline and iso-octane fuel.

**Figure 56.**
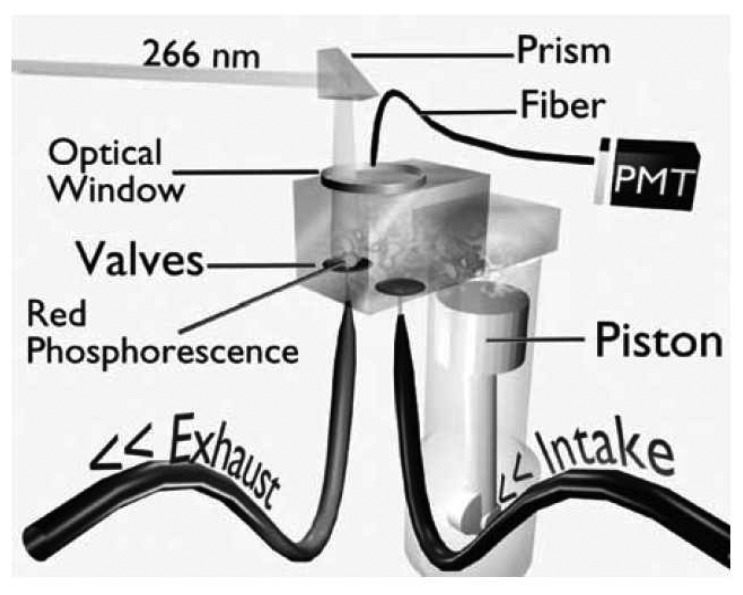
Schematic of temperature measurement inside an IC engine [86].

A thermographic phosphor was bonded onto the values using commercial binder. A fourth harmonic Nd:YAG (266 nm) was used to excite the phosphor through a quartz window. The emission signals were collected through a quartz fibre and digitised using a PMT and a fast oscilloscope (Figure 56). The lifetime decay response was used to determine temperature.

The success of single point temperature measurements was later extended to provide 2D thermal maps of engine walls, values and piston. A direct injection stratified charge(DISC) engine was used to provide such thermal maps [89]. An ICCD camera with a stereoscope and two interference filters (632 nm/657 nm) was used to image emissions at those wavelengths [86]. The intensity ratio approach was used to provide temperature data using pre-calibrated data.

**Figure 57.**
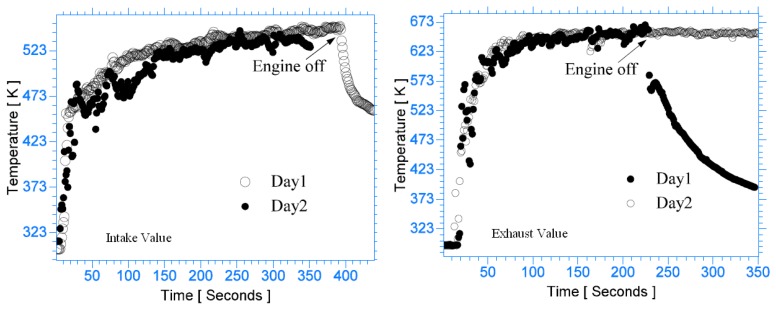
Temperature of the intake and exhaust values measured using thermographic phosphors [86].

**Figure 58.**
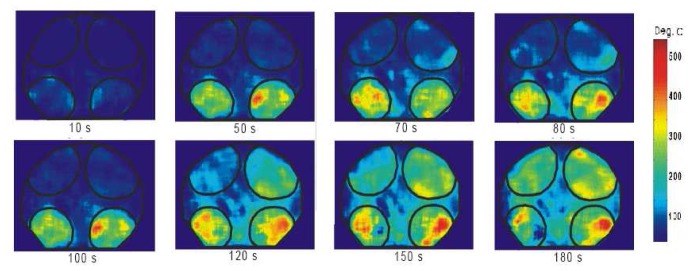
Temperature images of the intake(upper quadrant) and exhaust values(lower quadrant) measured using thermographic phosphors [86].

Both results, shown in Figure 57 and Figure 58 illustrate the exhaust values start to heat up earlier than the intake value, and also reaches a higher temperature. This is due to new fresh air entering the intake value allowing it to be cooled. In addition, the exhaust value also experiences burned gases from the combustion chamber that heat up the value [76].

### 11.7 2D Gas Temperatures

The thermographic phosphor method has recently been used to measure gas temperatures. Hasegawa *et al.* [36] investigated the intensity ratio of YAG:Dy for such measurements with the ultimate aim of measuring the gas temperatures in an operational engine.

**Figure 59.**
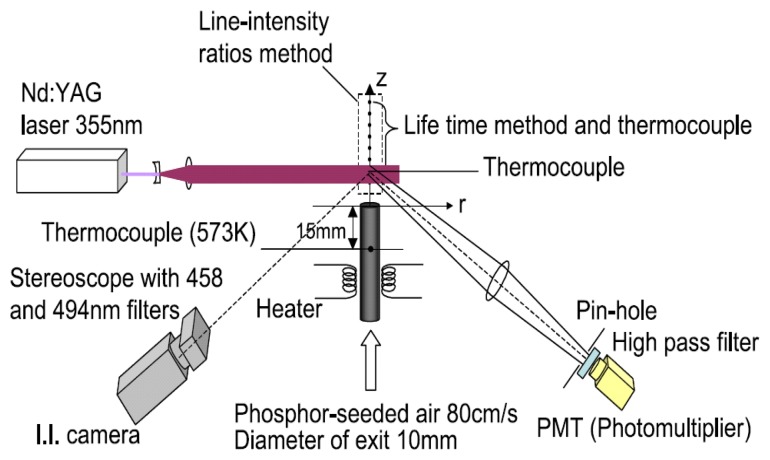
Experimental setup for temperature measurement for steady gas flow [36].

A static calibration, followed by a steady flow validation, was conducted before actual engine tests. In the calibration procedure, an imaging stereoscope and two band pass filters were used in front of an ICCD. The lifetime of the phosphor was also detected using a PMT to optimise the gate times for the ICCD. In gas flow validation experiments, phosphor-seeded air was passed through a 10 mm diameter exit at a velocity of 80 cm/s. A temperature-controlled heater was used to heat the flow to 573K 15 mm below the tube exit. A schematic is shown in Figure 59.

**Figure 60.**
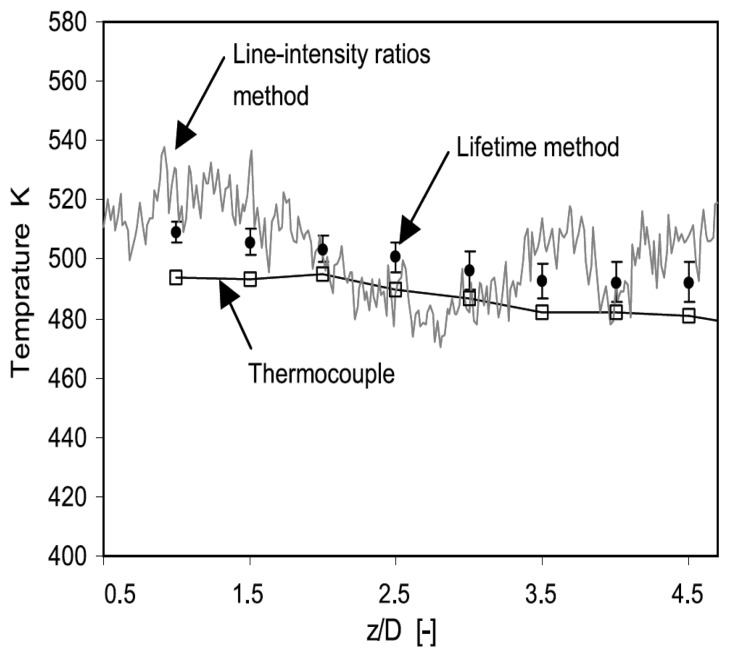
Gas temperature caparisons between thermocouple readings, lifetime method and intensity ratio method [36].

Figure 60 compares three different methods of measurement. The thermocouple readings were lower, as expected, since its intrusiveness perturbs the flow field with ambient room temperature, decreasing its temperature. According to Hasegawa *et al.* [36], the fluctuations in the intensity ratio method are mainly due to heterogeneity of gas seeding, causing a temperature error of around 5%.

During engine tests, a four cylinder diesel engine running at 1,200 RPM was used. A 355 nm Nd:YAG laser was used at 80 mJ/pulse and a homebuilt seeding device was used to obtain homogenous seeding. According to the authors, the intrusion of the phosphor caused an average drop in temperature by 2%. It was shown that phosphor thermometry could be used to measure temperatures of un-burnt gas flows. In turbulent combustion conditions inside the engine, phosphor temperature data agreed well within 5% error with the thermodynamic calculated data. However, chemi-luminescence effects caused measurements to be restricted after 10º after top dead centre. Further details on this can be found in the reference.

### 11.8 Droplets and Spray thermography

**Figure 61.**
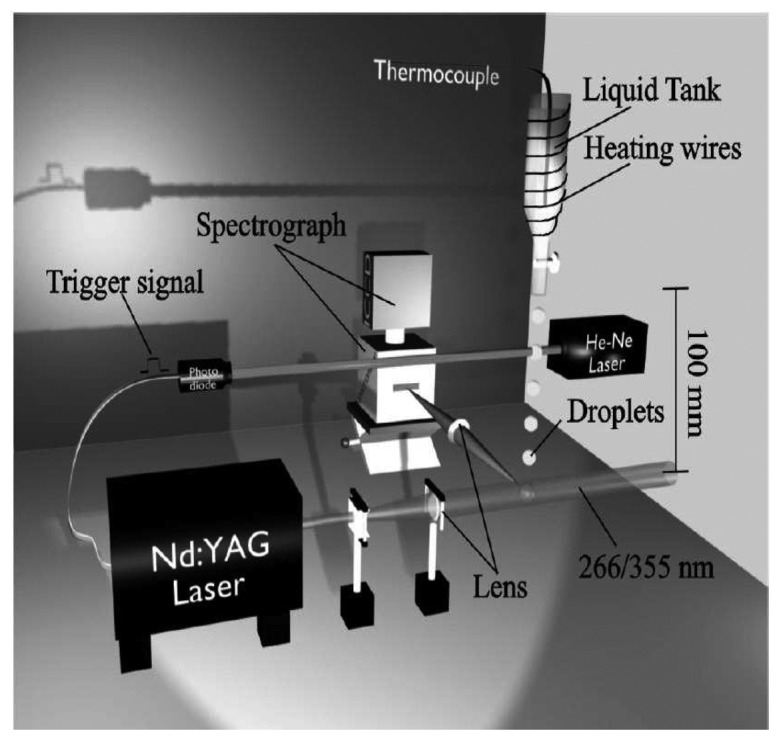
Experimental set up for droplet temperature measurement using thermographic phosphors [86].

Laser induced phosphoresce from thermographic phosphors was used to measure temperature of single falling droplets [90]. The droplets were excited using 266 nm Nd:YAG. The resulting emissions were evaluated spectrally, using a stereograph and Mg_3_FGeO_4_:Mn phosphor; and temporally, using a PMT and La_2_O_2_S:Eu phosphor. Phosphor (1%, by weight) was added to the liquid under investigation. A Nd:YAG laser was triggered when a droplet crossed a He-Ne beam. The setup is shown in Figure 61. The droplets were approx 3-4 mm in diameter. The temperature of the liquid was controlled using a thermocouple and heating wires around the container.

**Figure 62.**
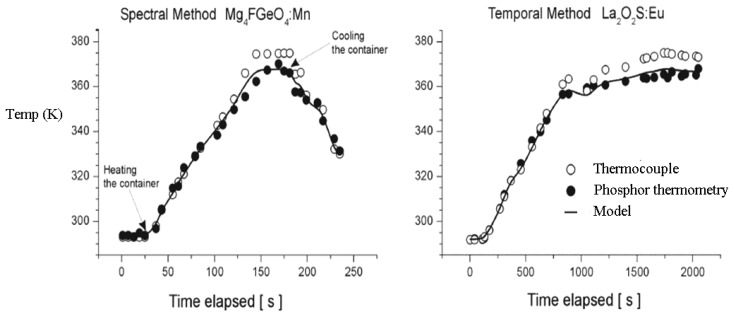
Temperature comparisons from thermocouple values, predicted model values, and actual phosphor measurements [86].

The results for both spectral and temporal methods are presented in Figure 62. The phosphor determined temperatures correspond well with the thermocouple readings from inside the tank. The thermocouple readings do not take into consideration the cooling and heat exchange to the surrounding air after it has left the nozzle.

To compensate for this, a model from Kinciad and Longley [91] was used. The results from the phosphors agreed very well to this with deviations less than 1%. Although the temperature of the droplets is of relatively low temperature, it does demonstrate the principle and may be used to measure much higher temperatures.

**Figure 63.**
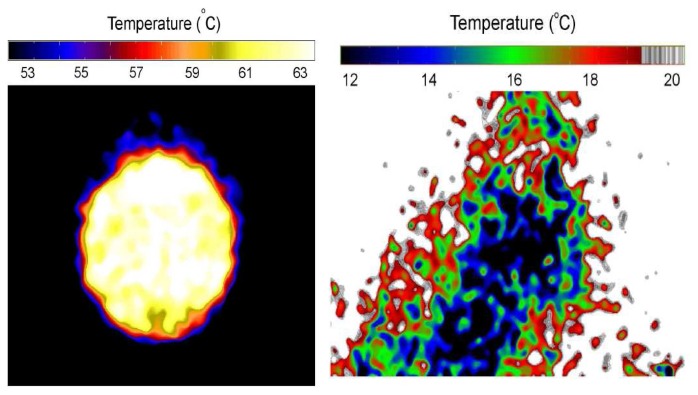
Examples of 2D droplet (left) and spray (right) thermometry using lifetime imaging [86].

The same methodology was extended to form 2D thermal measurements of droplets using fast framing ICCD cameras and decay lifetime imaging. The temperature at each pixel was evaluated using calibration procedure of lifetime against temperature. The technique was first applied to free falling water-based droplets, then to a suspended droplet in an ultrasonic levitator [92], and later to sprays [93]. Figure 63 illustrates some example of results that were obtained. Brubach *et al.* [94] also performed 2D spray and droplet thermometry using the intensity ratio approach using a Mg_4_GeO_5.5_F:Me thermographic phosphor. Further details can be found in the references.

### 11.9 Supersonic Combustor experiments

**Figure 64.**
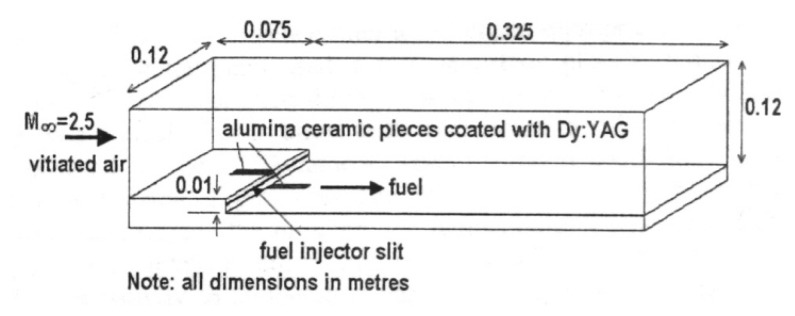
Schematic of a supersonic combustor test using thermographic phosphors [33].

Kontis [33] investigated surface transfer measurements inside a supersonic combustor. The experiment was conducted on a blown-down design supersonic wind tunnel. The run time was approximately 8 s with 4 s of steady state flow conditions at nominal values. The schematic of the test section can be seen in Figure 64 with further details explained in the reference. Hydrogen fuel was injected through a slit located along the back surface of the step. Two alumina ceramic pieces were secured using a high temperature adhesive both upstream and downstream of the step. These were coated with a thin layer (<50 µm) of YAG:Dy. The total thickness of the phosphor + ceramic was approx 0.01 m. Frequency tripled Nd:YAG laser (355 nm) was used to excite the phosphor after 1 s, allowing time for hydrogen fuel combustion. Emission profiles were then recorded every second using the intensity ratio method.

Due to the short run time, the surface under consideration did not have sufficient time to attain an equilibrium temperature. Transient techniques could be employed to measure heat flux. A one-dimensional unsteady heat transfer conduction was employed for both the phosphor layer and alumina-zirconia ceramic substrate.

The experimental results were then compared with computational simulations. Taking uncertainty factors into consideration, detailed in the reference, the overall uncertainty for heat flux determination was in the region of ±5%, which compares well to conventional transient techniques, such as thin-film or thermocouple gauges.

### 11.10 Hypersonic Wind tunnel testing

Advances in image processing and optical sciences have made the luminescent coating technique practical for aerodynamic wind tunnel testing. The fundamentals of aerodynamic testing with organic luminescent coatings (PSPs and TSPs) are well documented [15, 95]. Hubner *et al.* [96] reports the use of such paints to obtain heat transfer measurements at Mach 11.1 with the temperature ranging from 270K to a maximum of 400K. However, at higher temperatures, organic TSP became completely quenched. At higher temperatures, the use of thermographic phosphors would be more appropriate.

NASA Langley Research Centre has been using the relative-intensity two-colour phosphor thermography system for at least 15 years. It has become a standard technique to measure temperature and flux. An UV illuminated phosphor coated model is exposed to the wind tunnel flow. Subsequent emissions from two wavelengths are observed and converted to surface temperature maps using pre-calibrated data. With temperature maps acquired at different times during the run, global heat transfer images can be computed, assuming an one-dimensional semi-infinite heat conduction model. Scaled hypersonic models of X-33 [97], X-34 [98], X-38 [99, 100] and X-43 [101] have all been tested in wind tunnels using phosphor thermometry at Mach 6 and 10 in air, and some have been tested at Mach 20 in Helium [97]. Further details can be found in the references.

According to NASA, the primary advantage of thermographic phosphors is the global resolution of the quantitative heat transfer data which can be used to identify heating footprints of complex, 3D flow phenomena, including transitional fronts, turbulent wedges and boundary layer vortices, that are extremely difficult to resolve using discrete measurement techniques. In addition, the technique does not need corrections that are required for infrared thermometry.

**Figure 66.**
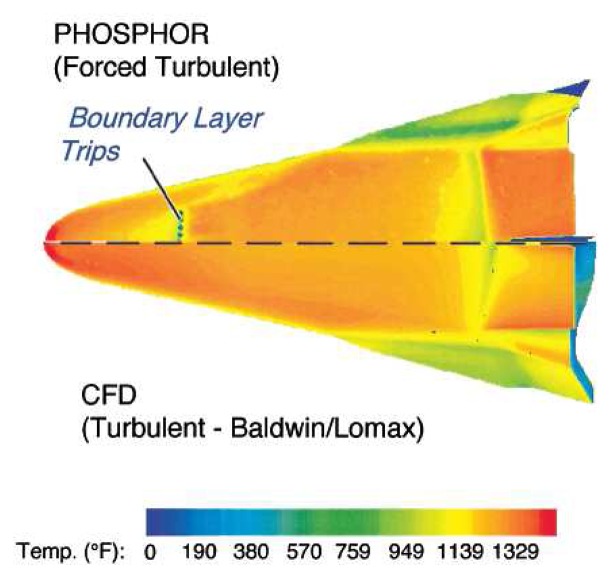

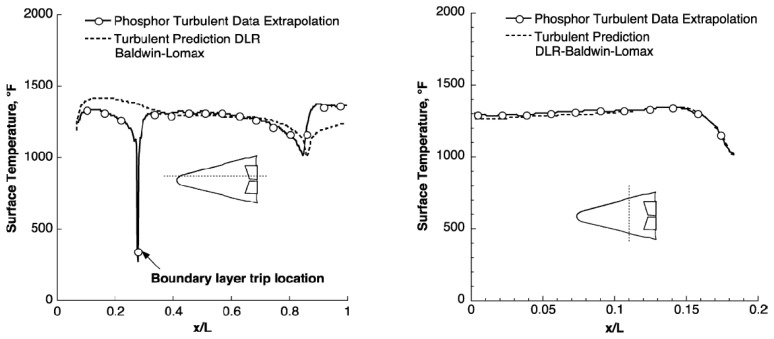
Comparison of extrapolated turbulent experimental data with predicted CFD data. Taken from Horvath *et al.* [100].

According to Hovarth *et al.* [97], measurement accuracy via phosphor thermometry is believed to better than around 8%, and the overall experimental uncertainty in heating data due to all factors is estimated to be around 15%, including uncertainties in the in the thermo-physical properties of the ceramic model. This is somewhat similar to that of thin films. Repeatability was found to be generally better, around 4% [100]. Merski [102] notes that the total uncertainties associated with the phosphor technique is shown to be approximately 7–10% in NASA's 31-Inch Mach 10 Tunnel and 8–10% in the 20-Inch Mach 6 Tunnel. A comparison with thin film measurements showed phosphor data to be within 7% and agreed better than CFD predictions. Figure 66 illustrates an example of the match between experimental phosphor data and predicted CFD data.

The phosphor technique provides a wealth of information critical to the design of thermal protection systems for applications involving engine design, re-entry vehicles, missiles and supersonic and hypersonic transport. Further research and work with phosphor thermometry at hypersonic speeds is planned at the University of Manchester in the near future.

### 11.11 Smart Thermal Barrier Coatings

Thermal Barrier Coatings (TBCs) provide thermal protection. They are usually found in very hot regions of a gas turbine engine. TBCs consist of a thin bond coat and an insulating layer, usually YSZ. They have a thickness in the order of 250 µm. TBC can be modified to behave like thermographic phosphors[103], exhibiting temperature dependable properties. The advantage of this is that no additional phosphor layers are required for temperature measurement. This concept was first proposed by Choy, Feist and Heyes [104].

YSZ:Eu and YSZ:Dy have been investigated both in powdered form and various forms of vapour depositions. Powdered YSZ:Eu response was observed with a dynamic range of 50-800ºC with a repeatability of ±0.1% using the lifetime method. YSZ:Dy was investigated using the intensity ratio method and was calibrated through a temperature range of 300-900K. This showed a repeatability of data around ±0.6% [34].

**Figure 67.**
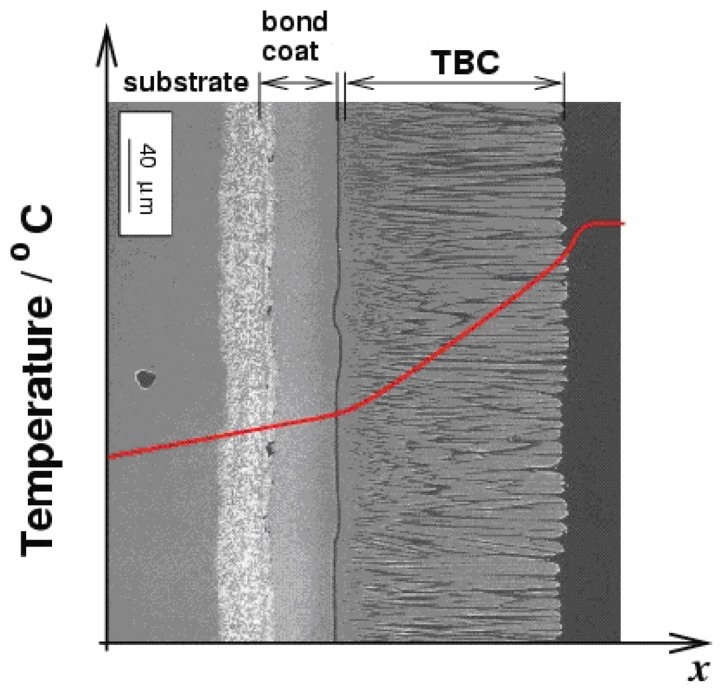
Temperature variation across the section of a TBC deposited on a substrate [105].

Using this methodology, it is possible to measure the temperature of the TBC. However, at high temperatures, there is a huge temperature gradient across the TBC, with temperatures being 200°C higher than that of the actual substrate. Figure 67 illustrates typical temperature variation across a TBC that is attached to a turbine blade experiencing high temperature flows. The actual substrate temperature is important for designers because it is this that determines material failures.

**Figure 68.**
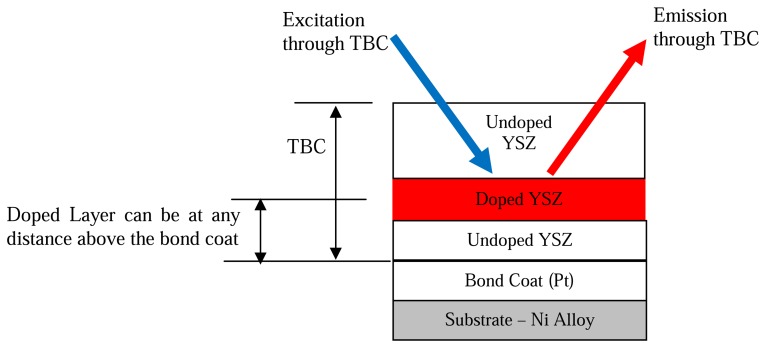
Phosphor thermometry at different distances though a YSZ-TBC.

Thermographic TBC may be created from multi-laminar construction layers as illustrated in Figure 68. With this arrangement, discreet points in the coating could be measured, including the temperature of the bond coat. The actual doped YSZ could be sandwiched in between the undoped YSZ allowing temperature measurement at various distances through the TBC. This information could be used to determine the health of thermal barrier coatings. Southside Thermal Sciences, UK are further developing this concept.

### 11.13 Galvanneal Process

**Figure 69.**
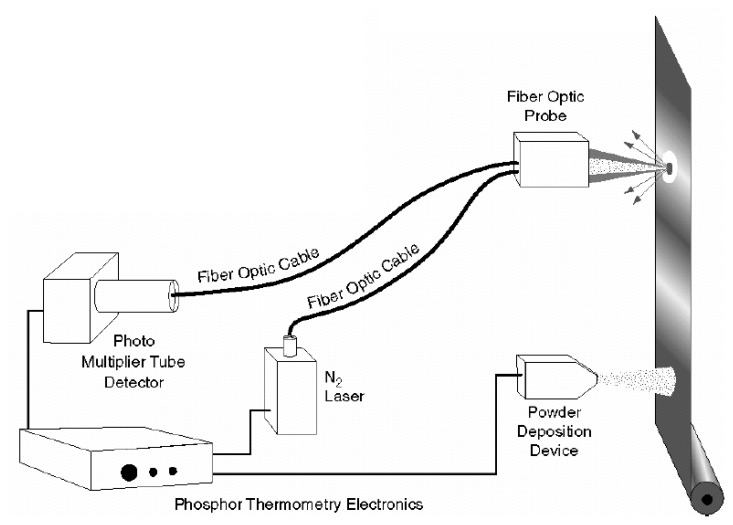
Schematic of the Galvanneal temperature measurement system [107].

The protective galvanneal process involves the dipping and heating of steel into molten zinc until the iron and zinc atoms form an alloy on the surface. The metals surface temperature may vary in the furnace, causing product quality and non-uniformity problems. Researchers at the ORNL developed a measurement system, based on the lifetime decay mechanism of phosphor thermometry to strictly control surface temperature, enabling the production of uniform, high-quality galvanneal steel [106]. The system, shown in Figure 69, includes a computerized phosphor-deposition device that is used to dust phosphor powder on the steel sheet. A portable nitrogen laser (377 nm, 0.3 mJ/pulse, 30Hz) is used to excite the phosphor, and subsequent emissions are detected and analysed in real time to evaluate and control the surface temperature by adjusting furnace settings. In 1998, the system was successfully installed and tested at Bethlehem Steel's facility in Burns Harbor, Indiana.

## 12. Conclusions

The idea of using phosphors for temperature measurements dates back to 1938. The capture and analysis of fast pulses required very expensive and sophisticated instrumentation. Over the past few decades there have been many advances in science and technology that allowed the phosphor technology to flourish and reach newer application areas. The fundamental principles of luminescence and phosphor thermometry were presented and various intensity, temporal and spectral approaches were discussed. Various other factors affecting the luminescence process were also discussed.

Phosphor thermometry is largely immune from errors common in pyrometry, such as emissivity and sensitivity to stray light. However, it requires bonding to the surface of interest, causing intrusiveness that can become relevant in complex situations. There is also an upper temperature limit due to increasing blackbody radiation and generally reducing phosphor signals at higher temperatures.

Recent developments and applications demonstrate phosphor thermometry being very flexible and successful in measuring temperatures in many different applications areas ranging from gas turbine measurements, internal combustion engine piston and value measurements, pyrolysis studies, to supersonic and hypersonic wind tunnel experiments. Apart from surface measurements, the technique has also been extended to measure temperatures of droplets, sprays and gases.

## Figures and Tables

**Figure 2.**
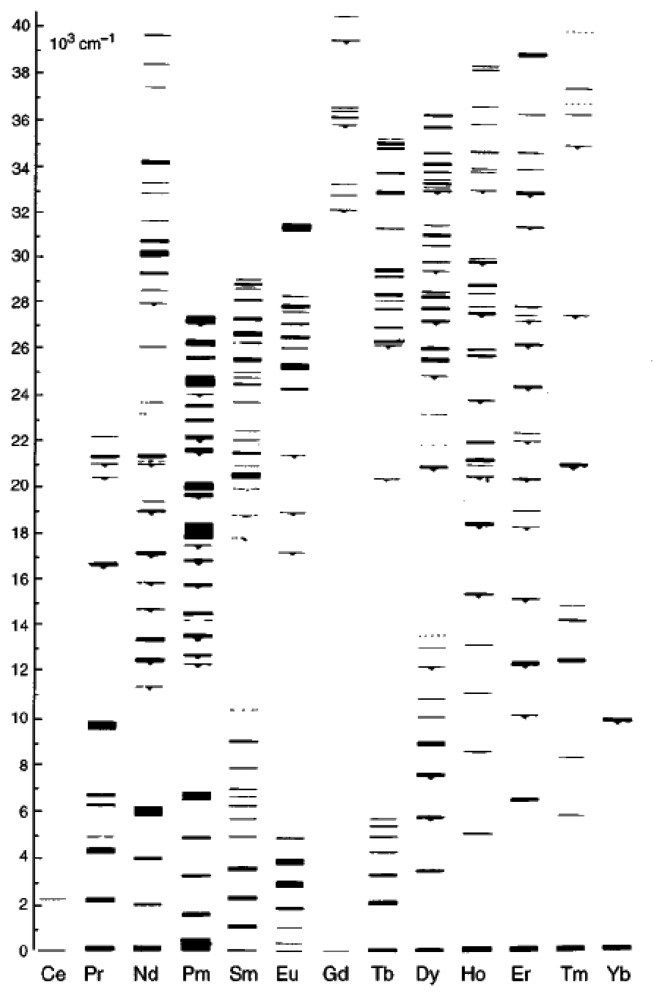
Energy level diagrams for some various rare earth materials. Taken from Allison and Gillies [2].

**Figure 4.**
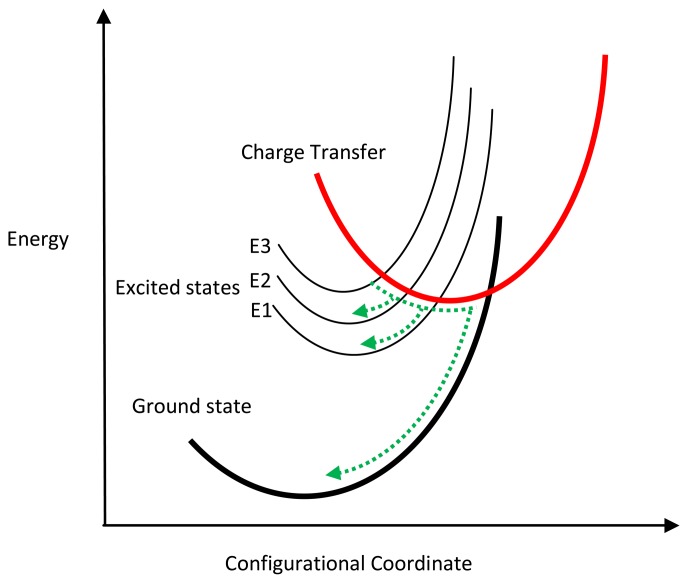
Configuration co-ordinate diagram showing the effect from the charge transfer state (CTS) curve.

**Figure 8.**
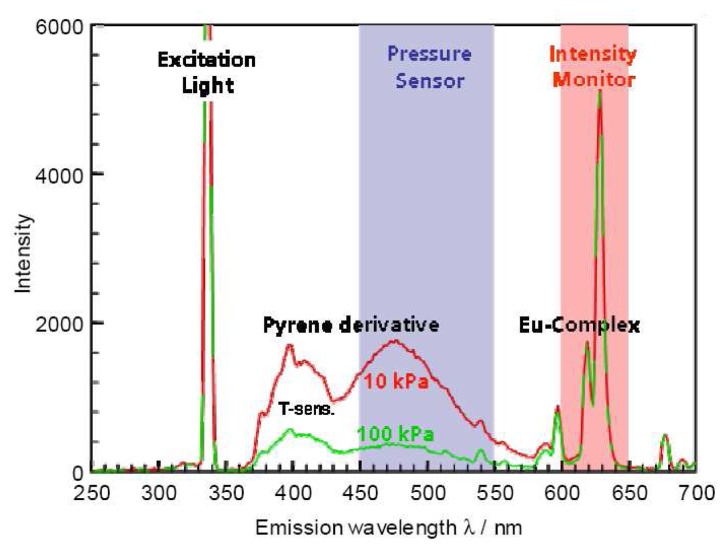
Typical emission spectrum of a typical binary PSP paint.

**Figure 19.**
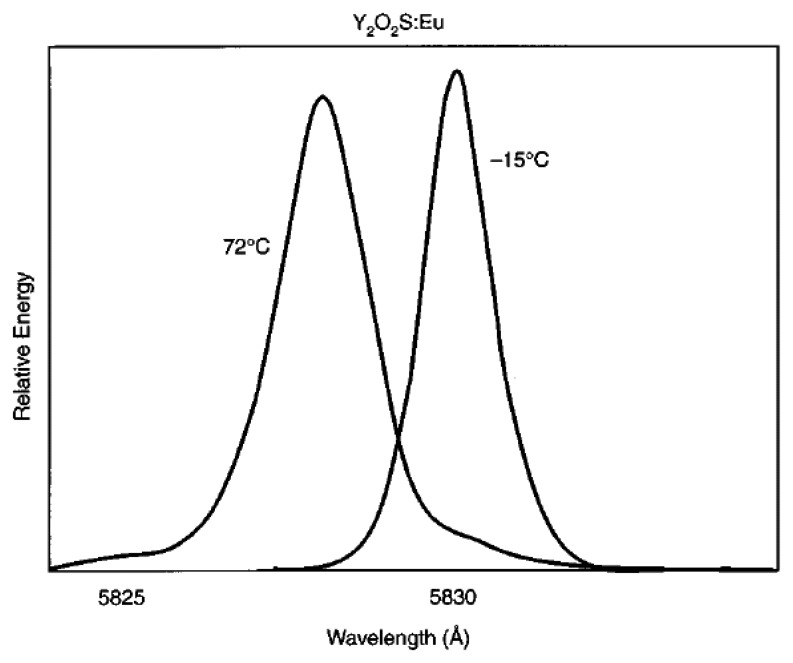
Emmsion lineshift and linewidth variation with temperature [2].

**Figure 29.**
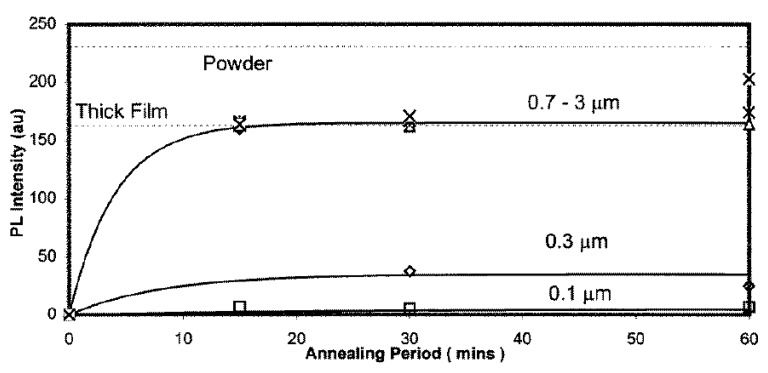
Intensity of thin coatings (0.1 µm-3 µm) after annealing at 1,200ºC compared to thick film (10 µm) and powdered Y_2_O_3_:Eu phosphor [60].

**Figure 40.**
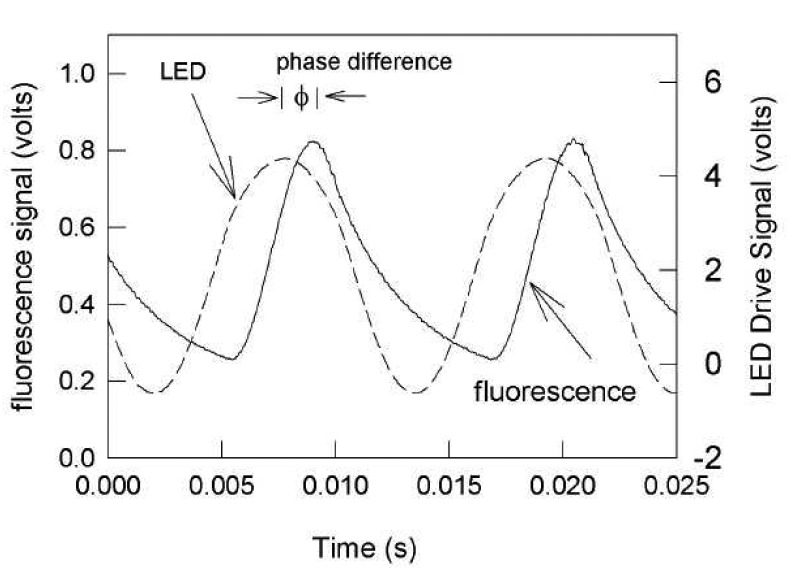
Sinusoidal wave used to excite phosphor. Taken from Allison *et al.* [42].

**Figure 41.**
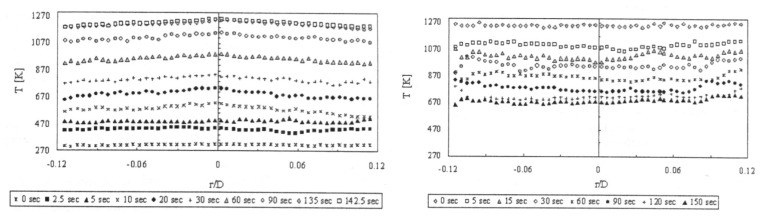
Centre region radial temperature profiles during a) jet impingement; b) cooling [32].

**Figure 46.**
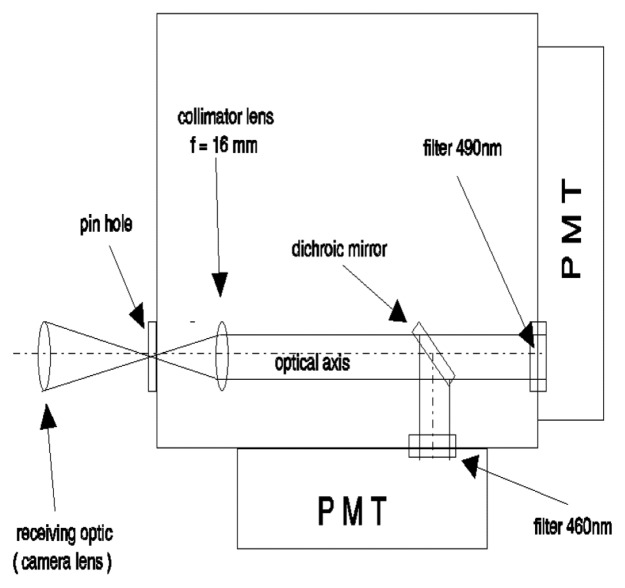
Modified system for simultaneous measurements of the intensity ratio and lifetime decay response modes [84].

**Figure 52.**
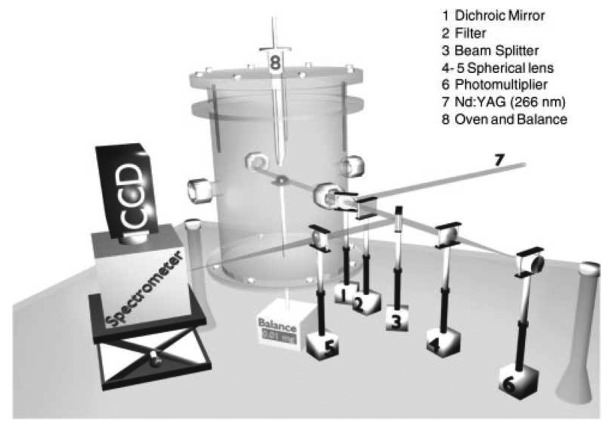
Schematic to study the pyrolysis of construction materials [87].

**Figure 65.**
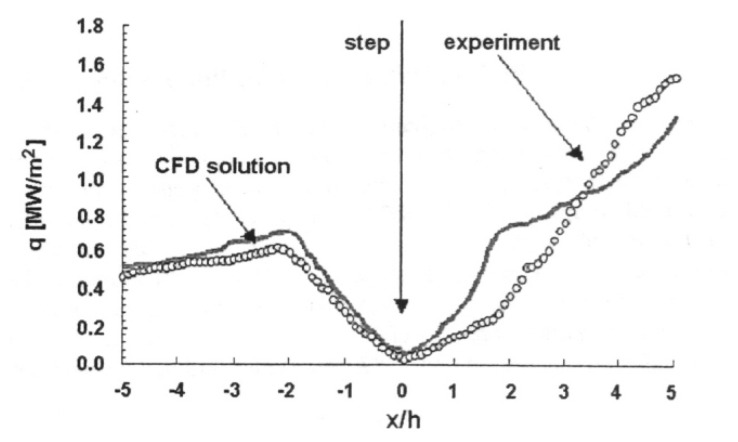
Heat flux comparisons along the centreline of the fuel injector side [33].

**Table 1.** Examples of various types of luminescence.

	**Energy Supplier:**	**Examples**

Chemi-luminescence	Chemical reactions	Glow in the dark plastic tubes, emergency light
Bio-luminescence	A form of chemi-luminescence where is the energy is supplied by living organisms.	Fireflies, glowworms
Electro-luminescence	Electric current	Certain watch displays e.g. Indiglo™
Cathode luminescence Radio-luminescence	Electron beam Nuclear radiation	CRT, televisions, Old glow in dark paints
Mechanoluminescence	Is light emission resulting from any mechanical action on a solid	
Triboluminescence	Some minerals glow when rubbed or scratched	Quartz crystal.
Fractoluminescence	Caused by stress that results in the formation of fractures.	
Sonoluminescence	The emission of short bursts of light from imploding bubbles in a liquid when excited by sound.	
Photoluminescence	Light energy. Commonly UV or visible light. Also includes laser induced fluorescence.	Phosphors, pressure sensitive paints.

**Table 2.** Summary of typical process times from excitation to emission.

**Transition Example**	**Process**	**Rate**	**Typical Timescale**

S_o_ → S_1_	Excitation, Absorption	k(e)	Femtoseconds, 10^−15^ s
	Internal Conversion	k(ic)	Picoseconds, 10^−12^ s
	Vibrational Relaxation	k(vr)	Picoseconds, 10^−12^ s
S_1_ → S_0_ (radiative)	Florescence	k(f)	Typically less than 10^−8^ s
S_1_ → S_0_ (non radiative)	Quenching and other non radiative processes	k(nr), k(q)	10^−7^ – 10^−5^ s
S_1_ → T_1_	Intersystem Crossing	k(pt)	10^−10^ – 10^−8^ s
T_1_ → S_0_	Phosphorescence	k(p)	10^−3^ – 100 s (earlier literature) > 10^−8^ s (recent literature)

**Table 3.** Elements in the a) lanthanide series; b) transition metals series.

**a) Lanthanides (rare earth ions)**		**b) Transition Metals**			
Ce	Cerium	Sc	Scandium	Cd	Cadmium
Pr	Praseodymium	Ti	Titanium	Hf	Hafnium
Nd	Neodymium	V	Vanadium	Ta	Tantalum
Pm	Promethium	Cr	Chromium	W	Tungsten
Sm	Samarium	Mn	Manganese	Re	Rhenium
Eu	Europium	Fe	Iron	Os	Osmium
Gd	Gadolinium	Co	Cobalt	Ir	Iridium
Tb	Terbium	Ni	Nickel	Pt	Platinum
Dy	Dysprosium	Cu	Copper	Au	Gold
Ho	Holmium	Zn	Zinc	Hg	Mercury
Er	Erbuim	Y	Yttrium	Rf	Rutherfordium
Tm	Thulium	Zr	Zirconium	Db	Dubnium
Yb	Ytterbium	Nb	Niobium	Gg	Seaborgium
Lu	Lutetium	Mo	Molybdenum	Bh	Bohrium
		Tc	Technetium	Hs	Hassium
		Ru	Ruthenium	Mt	Meitnerium
		Rh	Rhodium	Uun	Ununnilium
		Pd	Palladium	Uuu	Unununium
		Ag	Silver	Uub	Ununbium

**Table 4.** Overview of the limitations of the temperature measurement techniques in gas turbines. Taken from Feist *et al.* [22].

**Method**	**Disadvantage/Limitation**
Thermocouples	Intrusive Limited number Costly installation Bonding to ceramic surfaces Electromagnetic Interference Difficult to use on rotating components
Thermal Paints	Intrusive Time consuming (calibration and experiment) Irreversible Discrete values and poor resolutionCostly
Pyrometry	Sensitive to stray light Changes in emissivity Translucency of ceramic coatings Cleanness of optics
Thermographic Phosphor	Decreasing signals with increasing temperatures Bonding of the phosphor Phosphor coating can be intrusive

**Table 5.** Comparison of the ‘conventional two camera’ approach and the ‘filter wheel approach’ detection for the two-mode intensity method.

	Two camera	Filter Wheel + Single camera
Schematic	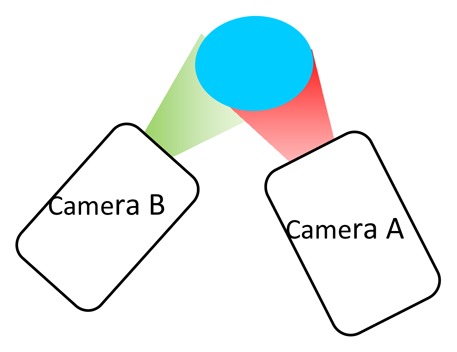	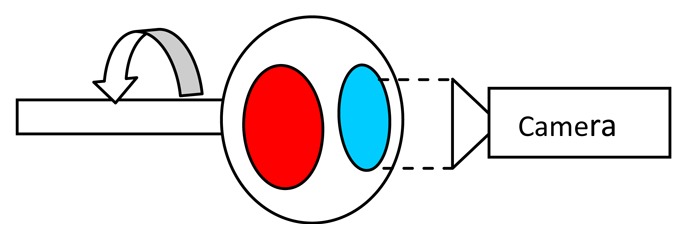
Signal capture	This system measures signals simultaneously.	This system measures both signals sequentially. Software is used separates out individual signals.
Alignment between images	Physical 3D alignment is required and errors may be induced.	The same camera and its position can eliminate many errors caused by alignment and CCD defects.
Mechanics	No moving parts	Reliable mechanical parts are required with repeatability to enable good signal separation.
Other	Flat field correction is required.	

**Table 6.** Effect of increasing temperature on the probability of radiative (P_r_) and non-radiative (P_nr_) decay.

**Equation**	**Effect if the k_nr_ value (or temperature) is increased**

$\begin{array}{c} \lambda =k_{r}+k_{\text{nr}}\\ \tau =\frac{1}{\lambda } \end{array}$	λ (the decay rate constant) will be increased. Hence, the decay lifetime of the transition will be decreased
$P_{r}=\frac{k_{r}}{k_{r}+k_{\text{nr}}}$	If the k_nr_ term is increased, the probability of radiative transition will be decreased. If the temperature is very high, this probability will yield to zero. (impossible)
$P_{\text{nr}}=\frac{k_{\text{nr}}}{k_{r}+k_{\text{nr}}}$	If the k_nr_ term is increased, the probability of non-radiative transition will increase, yielding to 1 (certainty) at high temperatures.

**Table 7.** Comparison of various high temperature chemical binders [59].

**Binder**	**Composition**	**Max temp of both survivability and observed fluorescence. (ºC)**

Sperex SP115	Silicone Resin	About 1,000
Sauerisen thinning	Soluble sodium silicate	1,204
liquid	Water based	
ZYP – BNSL	Glassy carbon and magnesium aluminium silicate	
ZYP – HPC	Alcohol and acetone based magnesium aluminium silicate water based	1,500
ZYP – LK	75% SiO2, 20% K2O, 5% LiO2. Water based	1,100
ZYP-LRC	Water based	Tested successfully up to 1,400
ZYP – ZAP	water-alcohol-based binder	Up to 1,600 (YAG:Dy)
Coltronics Resbond	791, 792 Silicate Glass, 793 Silica Oxide 795 -Alumina Oxide	up to 1,600

**Table 8.** Comparison of different light detectors.

	**Standard Photo-diodes**	**Avalanche Photodiode s APD**	**Conventional Photo Multiplier Tubes (PMTs)**	**Micro-channel plate PMT ( MCP-PMTs )**	**Si Photo-multipliers (SPM, SiPM)**

**Gain**	None	Low gain (x10-300)	High Gain (10^6^)	High Gain (10^6^) Typical = 5 ×10^5^	High Gain (10^6^) Much higher gains than APDs. Same region as PMTs.
**Bias V**		High (800V)	High (kV)		Low (30V)
**Sensitivity**	Typically < 1 A/W	25 A/W @ 520 nm30 A/W @ 1,064 nm	40,000 A/W @ 520 nm(SensL)[67]Sensitivity: 110 uA/lmAnode sens:500 A/lm (nominal);2,000 A/lm (max)	Very highCathodesensitivity>1,200 uA/lm (min), 1,500 (typical)(Burle)[62]	60,000 A/W @ 520 nm (micro)130,000 A/W @520 nm (mini)1,000 A/W @ 1,064nm
**Response to excess light**	Some PIN damaged	Some APDs are damaged	Damage		Tolerant
**Area**	Small	Small	Large diameters-e.g. 46mm.Arrays are impossible		Small (1×1 mm^2^)large areas up to 9mm^2^ available.Building larger arrays is possible.
**Rise/Fall Times -response**	Trise = 0.1microseconds	Can be operated at 2,000 MHz. (therefore ns)	Rise time: 1 ns.	Faster thanPMTs.100 picoseconds	<5ns
**Quantum Efficiencies**	Higher than PMTs	Higher than PMTs	20-30% at peak	>20% at peak	40% @ 520 nm at peak
**SNR (signal to noise)**	Low	Low.	HighPMTs offer a higher gain, larger detection area and superior SNR compared to APDs		High –SNR to be similar, and in some cases better than PMTs.
**Other notes**	Robust	Robust	Fragile, affected by magnetic, electromagnetic interference.	Excellent for pulsed light.	Robust
